# Risk of Inflammatory Bowel Disease After Anti-CD20 Treatment in Multiple Sclerosis

**DOI:** 10.1001/jamaneurol.2026.3439

**Published:** 2026-10-22

**Authors:** Sandra Kallin, Anders Svenningsson, Ola Olén, Charlotte Hedin, Jan Lycke, Peter Alping

**Affiliations:** 1 Department of Clinical Sciences, Karolinska Institutet Danderyd Hospital, Stockholm, Sweden; 2 Department of Neurology, Danderyd Hospital, Stockholm, Sweden; 3 Division of Clinical Epidemiology, Department of Medicine Solna, Karolinska Institutet, Stockholm, Sweden; 4 Sachs’ Children and Youth Hospital, Stockholm South General Hospital, Stockholm, Sweden; 5 Department of Medicine Solna, Karolinska Institutet, Stockholm, Sweden; 6 Centre for Digestive Health, Department of Gastroenterology, Dermatovenereology and Rheumatology, Karolinska University Hospital, Stockholm, Sweden; 7 Institute of Neuroscience and Physiology, Sahlgrenska Academy, Gothenburg University, Gothenburg, Sweden; 8 Sahlgrenska University Hospital, Department of Neurology, Region Västra Götaland, Gothenburg, Sweden

## Abstract

**Importance:**

Anti-CD20 biologics are widely used as disease-modifying therapy (DMT) for relapsing-remitting multiple sclerosis (RRMS), but recent reports have raised concerns about an increased risk of inflammatory bowel disease (IBD) during treatment. This study aimed to assess treatment-associated IBD risk in individuals with RRMS.

**Objective:**

To compare the risk of new-onset IBD across different therapies for RRMS and relative to the general population.

**Design, Setting, and Participants:**

This was a population-based cohort study conducted in Sweden. Through the Swedish MS register, initiations of rituximab, natalizumab, dimethyl fumarate, and fingolimod were identified between January 1, 2011, and December 31, 2023, in individuals with RRMS aged 18 to 55 years with at least 10 years of lookback time (5 years for dispensed drugs) and no evidence of previous IBD. These initiations were matched with general population comparator episodes selected from the Swedish population.

**Exposures:**

Rituximab, comparator DMTs (natalizumab, dimethyl fumarate, fingolimod), or general population comparator.

**Main Outcomes and Measures:**

New-onset IBD, defined as 2 or more records on separate dates with the *International Statistical Classification of Diseases and Related Health Problems*, *Tenth Revision*, codes K50 (Crohn disease), K51 (ulcerative colitis), and/or K52.3 (indeterminate colitis).

**Results:**

Among the 13 748 therapy episodes (6481 rituximab, 2976 natalizumab, 2767 dimethyl fumarate, 1524 fingolimod) in 9251 individuals (range in mean ages of the exposure groups, 35-38 years) and 61 065 matched general population episodes, 164 IBD events occurred during follow-up (40 with rituximab, 4 with comparator DMTs, and 120 in the general population). The crude incidence rate of IBD was 110 (95% CI, 76-147) per 100 000 person-years for rituximab, 13 (95% CI, 3-30) for the comparator DMTs, and 41 (95% CI, 34-48) for the general population. After adjustment for demographics and MS characteristics, the hazard ratio for the comparator DMTs vs rituximab was 0.10 (95% CI, 0.04-0.22). In the rituximab group, 62% of IBD events were classified as Crohn disease compared with 29% in the general population. The number needed to harm for rituximab vs the comparator DMTs was 131 (95% CI, 93-228) at 5 years, after full adjustment.

**Conclusions and Relevance:**

Results of this cohort study show that rituximab was associated with an increased risk of IBD compared with other modern MS therapies, after adjustment for differences in characteristics at therapy start. Clinicians and individuals with MS should consider IBD risk in treatment decisions and remain vigilant for gastrointestinal symptoms during anti-CD20 therapy.

## Key Points

**Question:**

Is anti-CD20 therapy associated with an increase in the risk of inflammatory bowel disease in individuals with multiple sclerosis?

**Findings:**

In this population-based cohort study of 13 748 therapy episodes in 9251 individuals with relapsing-remitting multiple sclerosis, the anti-CD20 therapy rituximab was associated with an increased risk of inflammatory bowel disease compared with other modern disease-modifying therapies.

**Meaning:**

Study results suggest that clinicians and individuals with multiple sclerosis should consider the risk of inflammatory bowel disease when making treatment decisions and remain vigilant for gastrointestinal symptoms during anti-CD20 therapy.

## Teaser

This cohort study investigates if anti-CD20 treatment is associated with an increase in the risk of inflammatory bowel disease in individuals with multiple sclerosis (MS).

## Introduction

Multiple sclerosis (MS) is a chronic autoimmune disorder of the central nervous system, most often following a relapsing-remitting disease pattern (RRMS).^1^ Anti-CD20 therapies, which deplete circulating CD20-positive lymphocytes, have proven to be among the most effective at reducing acute inflammatory activity in MS.^2^ The first anti-CD20 therapy investigated for MS, rituximab, was never taken to market for MS by its marketing authorization holder (Roche Registration GmbH), and newer alternatives (ocrelizumab, ofatumumab, and ublituximab) were later approved for MS.^3^ After its initial promising results and supported by extensive experience in rheumatoid arthritis, rituximab has been increasingly used off-label to treat MS and is now the most commonly used MS disease-modifying therapy (DMT) in Sweden.^7^

Despite an overall favorable safety profile, anti-CD20 treatment has been associated with an increased risk of severe infections,^8^ hypogammaglobulinemia,^10^ and reduced vaccination response.^12^ Additionally, postmarketing reports have indicated a potential increase in the risk of new-onset inflammatory bowel disease (IBD) with ocrelizumab,^14^ ofatumumab,^20^ and rituximab.^21^ Data from larger observational studies on anti-CD20–associated IBD in MS are lacking. To address this, we conducted a population-based cohort study using Sweden’s national registers to quantify and compare the risk of IBD in individuals with RRMS treated with different DMTs.

## Methods

In this population-based cohort study, we linked data from the Swedish MS register^24^ with national demographic and medical registers for individuals with MS and matched general population comparators (eMethods in Supplement 1 contains a detailed description of the setting and data sources). The study was approved by the Swedish Ethical Review Authority and was reported in accordance with the Strengthening the Reporting of Observational Studies in Epidemiology (STROBE) reporting guidelines. As this was an observational study, informed consent from participants was not required under Swedish law.

### Study Population and Exposure

We included therapy episodes with rituximab, natalizumab, dimethyl fumarate, and fingolimod, started between January 1, 2011, and December 31, 2023, in individuals with RRMS. This allowed for at least 10 years of lookback for inpatient and outpatient visits and 5 years of lookback for prescription drugs, as well as at least 2 years of potential follow-up for identifying outcome events, given the availability of the register data (data cutoff December 31, 2025). Additionally, each individual in the matched comparator population contributed general population episodes corresponding to the therapy episodes of their matched person with MS, using the same start dates (therapy initiation) and stop dates (therapy discontinuation) as for the index therapy episode (eMethods in Supplement 1). General population episodes that started after the person developed MS were excluded.

The unit of observation in this study was an exposure episode (ie, a therapy or general population episode). Each individual could contribute several exposure episodes, and the nonrituximab therapies were grouped together as comparator DMTs to increase the number of events per comparison group.

The exposure episodes were restricted to those in individuals aged 18 to 55 years at episode start, without prior IBD suspicion (based on *International Statistical Classification of Diseases and Related Health Problems, Tenth Revision* [*ICD-10*], and *Anatomical Therapeutic Chemical* [*ATC*] classification codes, listed in the eMethods in Supplement 1) and with at least 10 years of register data before episode start. Lookback time was counted from the latest of birth, immigration, or the start of register data. Participant race and ethnicity data are not available.

Each exposure episode was followed up until first IBD diagnosis, data cutoff, emigration, death, or episode stop date plus a 6-month extension period, whichever occurred first. The extension period was added to avoid misclassification of outcome status in cases where therapy was discontinued due to IBD symptoms before IBD diagnosis. General population episodes were additionally censored at the date of MS diagnosis, if the person developed MS.

### Outcome

New-onset IBD was identified through *ICD-10* codes from the National Patient Register, after episode start, in accordance with a previously validated definition, which was as follows: at least 2 observations on separate dates in inpatient or secondary outpatient care, with the code K50 (Crohn disease), K51 (ulcerative colitis), and/or K52.3 (indeterminate colitis).^25^ The date of the first code was used as the outcome date, and therefore, only this code had to fall within the follow-up period. We further classified the IBD outcome, based on the *ICD* codes registered for the first 2 observations, into Crohn disease (only K50 codes), ulcerative colitis (only K51 codes), or unclassified IBD (any other combination of K50, K51, and K52.3)^26^ (eMethods in Supplement 1).

### Covariates

Several potential confounders were identified in the time before episode start and adjusted for in the analysis. Demographic variables included age, sex, country of birth (Sweden or other), civil status (married or partner), annual income (mean over the previous 3 years, adjusted for inflation), education (primary, secondary, or higher education), and area of residence in Sweden. MS-specific characteristics were MS duration, year of therapy start, number of previous DMTs, number of previous relapses, Expanded Disability Status Scale (EDSS),^27^ Symbol Digit Modalities Test (SDMT),^28^ and Multiple Sclerosis Impact Scale (MSIS-29).^29^ Baseline scores were chosen as the score closest to episode start, from 1 year before to 1 month after, ignoring any score recorded within 30 days of a clinical relapse.

### Statistical Analysis

Baseline characteristics were tabulated, stratified by both exposure group and outcome status. The number of exposure episodes still at risk of the outcome was tabulated by year over the follow-up. The number of events, the number and proportion of each IBD subtype, and the total and median observed time were calculated for each exposure group. Incidence rates (IRs) were calculated as the number of events divided by the observed time. Hazard ratios (HRs) were estimated from Cox proportional hazards models with time since episode start as the time scale and rituximab as the reference. Because of the small number of outcome events in the nonrituximab groups, HRs were estimated only for the grouped comparator DMTs and for the general population, and penalization was used in the Cox regression as recommended for sparse data by Greenland et al (eMethods in Supplement 1).^30^ IRs and HRs were presented in both tables and forest plots. Survival curves were estimated using the Kaplan-Meier method. Finally, numbers needed to harm over time were estimated for rituximab from the survival curves as the inverse of the difference in cumulative incidence between rituximab and the comparator DMTs at each time point. Study data were analyzed using Python, version 3.14 (Python Software Foundation), and R, version 4.6.0 (R Foundation for Statistical Computing). Data management was performed with Polars (Python), imputation with MICE (R), Kaplan-Meier with lifelines (Python), and multinomial logistic regression with scikit-learn (Python). Penalized Cox regression was performed using a custom model purpose-built with JAX (Python).

#### Confounder Adjustment and CIs

All analyses were performed without confounder adjustment (crude), adjusting for demographic variables, and additionally adjusting for MS characteristics. Confounder adjustment was performed by inverse probability of treatment weighting, where weights were estimated as the inverse of the predicted probability of the observed exposure from a multinomial logistic regression, stabilized by the marginal probability of the observed exposure. Truncation of extreme weights was not required. 95% CIs were estimated using cluster bootstrapping (1000 replicates) and the percentile method, with the individuals with MS as the clusters (for both the therapy and general population episodes, preserving the matching).

#### Missing Data

Missing data were addressed by stochastic predictive mean matching, including all variables from the weighting model, as well as an indicator of the outcome and the Nelson-Aalen estimate of the cumulative hazard within each exposure group. To reduce complexity, the few observations with missing data among the demographic variables were excluded when these variables were used for adjustment, and imputation was performed only for MS characteristics (eMethods in Supplement 1).

#### Sensitivity Analysis

Several sensitivity analyses were performed to assess the robustness of the results given different assumptions (eMethods in Supplement 1). To address the differences in time on treatment, we used an ever-treated approach to follow-up, meaning that only the first occurrence of each exposure per person was included, and follow-up did not end at therapy discontinuation. To address potential misclassification of the outcome, we used a stricter definition of IBD, requiring at least 3 IBD listings with at least 1 year between the first and last. Finally, to account for changing treatment patterns over time, we restricted inclusion to a period with greater overlap in start year between the therapy groups.

## Results

We included a total of 13 748 therapy episodes (6481 rituximab, 2976 natalizumab, 2767 dimethyl fumarate, 1524 fingolimod), contributed by 9251 individuals (range in mean ages of the exposure groups, 35-38 years) with RRMS, and 61 065 general population episodes, contributed by 40 925 individuals without MS (Table 1 and eTables 1 and 2 in Supplement 1). Median (IQR) observed time was 5.5 (3.1-7.8) years for rituximab, 2.7 (1.2-6.3) years for the comparator DMTs, and 4.1 (1.9-7.4) years for the general population (Table 2 and eFigure 1 in Supplement 1). Median (IQR) time to IBD event was 2.9 (1.9-4.9) years for rituximab, 1.8 (1.1-2.5) years for the comparator DMTs, and 2.8 (1.2-5.8) years for the general population (eFigure 2 in Supplement 1). Of the IBD cases in the rituximab group, 30% discontinued treatment just before or within 2 years of first IBD diagnosis (eFigure 3 in Supplement 1).

**Table 1.** Baseline Characteristics at Exposure Start Stratified by Exposure Group

Variable	Rituximab	Natalizumab	Dimethyl fumarate	Fingolimod	General population
No.	6481	2976	2767	1524	61 065
Year of episode start, median (IQR)	2018 (2016-2021)	2016 (2013-2020)	2016 (2014-2018)	2013 (2012-2015)	2016 (2014-2020)
Age, mean (SD), y	38.4 (9.1)	34.8 (8.5)	38.2 (8.9)	38.0 (8.6)	37.8 (9.0)
Sex, No. (%)					
Female	4547 (70.2)	2227 (74.8)	2040 (73.7)	1051 (69.0)	43 801 (71.7)
Male	1934 (29.8)	749 (25.2)	727 (26.3)	473 (31.0)	17 264 (28.3)
Born in Sweden, No. (%)	5812 (89.7)	2741 (92.1)	2518 (91.0)	1384 (90.9)	52 636 (86.2)
Married/partner, No. (%)	2366 (36.5)	1009 (33.9)	1109 (40.1)	632 (41.5)	22 207 (36.8)
Yearly income, median (IQR), thousand SEK [US $]^a^	316.6 (123.4-469.8) [31.7 {12.3-47.0}]	270.8 (108.2-419.7) [27.1 {10.8-42.0}]	308.2 (145.7-449.9) [30.8 {14.6-45.0}]	271.3 (94.9-411.8) [27.1 {9.5-41.2}]	328.7 (142.0-464.6) [32.9 {14.2-46.5}]
Education, No. (%)					
Primary	520 (8.1)	259 (8.7)	209 (7.6)	128 (8.4)	5275 (8.7)
Secondary	2888 (44.7)	1346 (45.4)	1250 (45.3)	714 (47.0)	26 792 (44.2)
Higher	3051 (47.2)	1360 (45.9)	1302 (47.2)	677 (44.6)	28 508 (47.1)
Time since MS onset, median (IQR), y	4.9 (1.1-11.4)	3.3 (0.5-8.7)	4.0 (0.8-10.2)	7.4 (3.4-12.7)	NA
Previous DMTs, median (IQR), No.	1.0 (0.0-2.0)	1.0 (0.0-2.0)	1.0 (0.0-1.0)	1.0 (1.0-2.0)	NA
Previous DMTs, No. (%)					
Natalizumab	1938 (29.9)	517 (17.4)	151 (5.5)	507 (33.3)	NA
Fingolimod	703 (10.8)	286 (9.6)	100 (3.6)	91 (6.0)	NA
Rituximab	150 (2.3)	161 (5.4)	64 (2.3)	35 (2.3)	NA
Dimethyl fumarate	1161 (17.9)	300 (10.1)	291 (10.5)	109 (7.2)	NA
Previous relapses, median (IQR), No.	1.0 (0.0-2.0)	1.0 (0.0-2.0)	1.0 (0.0-2.0)	2.0 (1.0-3.0)	NA
EDSS, No. (%)					
Mild	2362 (79.0)	864 (75.0)	1098 (85.9)	554 (75.9)	NA
Moderate	530 (17.7)	233 (20.2)	150 (11.7)	144 (19.7)	NA
Severe	99 (3.3)	55 (4.8)	30 (2.3)	32 (4.4)	NA
SDMT, median (IQR)	53.0 (46.0-61.0)	53.0 (45.0-60.0)	53.0 (47.0-60.0)	53.5 (45.0-62.0)	NA
MSIS-29, median (IQR)					
Physical	1.5 (1.1-2.2)	1.6 (1.2-2.5)	1.4 (1.1-2.0)	1.5 (1.1-2.1)	NA
Psychological	2.1 (1.4-2.9)	2.3 (1.7-3.1)	1.9 (1.3-2.8)	2.0 (1.4-2.8)	NA
Area, No. (%)					
Stockholm	1791 (27.6)	435 (14.6)	473 (17.1)	275 (18.1)	12 993 (21.5)
East Middle Sweden	1104 (17.0)	405 (13.6)	468 (16.9)	211 (13.9)	9658 (16.0)
South Sweden	784 (12.1)	576 (19.4)	527 (19.1)	248 (16.3)	9080 (15.1)
North Middle Sweden	598 (9.2)	182 (6.1)	279 (10.1)	120 (7.9)	5212 (8.6)
Middle Norrland	296 (4.6)	103 (3.5)	71 (2.6)	44 (2.9)	2325 (3.9)
Upper Norrland	460 (7.1)	131 (4.4)	47 (1.7)	52 (3.4)	3047 (5.1)
Småland and the islands	407 (6.3)	159 (5.3)	251 (9.1)	190 (12.5)	4601 (7.6)
West Sweden	1039 (16.0)	984 (33.1)	650 (23.5)	383 (25.1)	13 389 (22.2)

Abbreviations: DMT, Disease-Modifying Therapy; EDSS, Expanded Disability Status Scale; MS, multiple sclerosis; MSIS-29, Multiple Sclerosis Impact Scale; NA, not applicable; SDMT, Symbol Digit Modalities Test; SEK, Swedish krona.

^a^ Conversion factor 1 Swedish krona = 0.10 US dollar.

**Table 2.** Observed Time, Events, Incidence Rates (IRs), and Hazard Ratios (HRs), Stratified By Exposure Group^a^

Group	No.	Person-years (median)	Events, No.	CD/UC/unclassified (%)	IR			HR		
					Crude	Demographics	MS characteristics	Crude	Demographics	MS characteristics
Rituximab	6481	36 217 (5.5)	40	25/6/9 (62/15/22)	110.4 (76.0-146.6)	128.2 (85.3-179.6)	134.3 (88.4-181.9)	NA	NA	NA
Comparator DMTs	7267	29 756 (2.7)	4	1/3/0 (25/75/0)	13.4 (3.3-30.1)	13.0 (1.9-28.1)	10.8 (2.2-24.1)	0.14 (0.05-0.31)	0.12 (0.04-0.26)	0.10 (0.04-0.22)
General population	61 065	295 112 (4.1)	120	35/67/18 (29/56/15)	40.7 (33.5-48.1)	41.9 (34.6-49.6)	NA	0.38 (0.27-0.56)	0.33 (0.23-0.52)	NA
Natalizumab	2976	11 097 (2.3)	2	1/1/0 (50/50/0)	18.0 (0.0-46.1)	15.1 (0.0-42.8)	13.8 (0.0-40.6)	NA	NA	NA
Dimethyl fumarate	2767	10 444 (2.6)	1	0/1/0 (0/100/0)	9.6 (0.0-36.8)	11.3 (0.0-40.8)	9.2 (0.0-27.9)	NA	NA	NA
Fingolimod	1524	8215 (4.3)	1	0/1/0 (0/100/0)	12.2 (0.0-37.4)	12.5 (0.0-38.8)	5.5 (0.0-35.7)	NA	NA	NA

Abbreviations: CD, Crohn disease; DMT, disease-modifying therapy; HR, hazard ratio; IR, incidence rate; MS, multiple sclerosis; NA, not applicable; UC, ulcerative colitis.

^a^ IRs per 100 000 person-years and penalized HRs with 95% CIs. Time since episode start as the time scale and rituximab as the reference. Comparator DMTs grouped natalizumab, dimethyl fumarate, and fingolimod. For the adjusted IRs, separate models were fitted with the comparator DMTs grouped and ungrouped (the rows starting with natalizumab, dimethyl fumarate, and fingolimod). HRs were not estimated for the ungrouped therapies due to sparse data. Demographics were adjusted for sex, age, country of birth (Sweden or other), civil status (married/partner), annual income, education, and area of residence in Sweden. MS characteristics were adjusted for MS duration, year of therapy start, number of previous DMTs, number of previous relapses, and baseline Expanded Disability Status Scale, Symbol Digit Modalities Test, and Multiple Sclerosis Impact Scale.

### Baseline Characteristics

Baseline characteristics differed between therapy groups (Table 1), with rituximab treatment initiated later in time and natalizumab at younger ages. The matched general population had a higher median annual income, but was otherwise broadly similar to the therapy groups. Missing data were less than or equal to 1.2% for demographic variables and 35% to 61% for MS characteristics (eTable 3 in Supplement 1).

### Events and IRs

We identified 164 incident IBD events as follows: 40 for rituximab, 4 for the comparator DMTs, and 120 for the general population, resulting in crude IRs of 110 (95% CI, 76-147), 13 (95% CI, 3-30), and 41 (95% CI, 34-48) per 100 000 person-years, respectively (Table 2 and Figure 1). After adjustment for demographics and MS characteristics, the difference in incidence rate between rituximab and the comparator DMTs increased. In the rituximab group, 25 of IBD events (62%) were classified as Crohn disease, 6 (15%) as ulcerative colitis, and 9 (22%) as unclassified IBD (Table 2), which was a higher proportion of Crohn disease compared with the comparator DMTs (25%) and the general population (29%).

**Figure 1.**
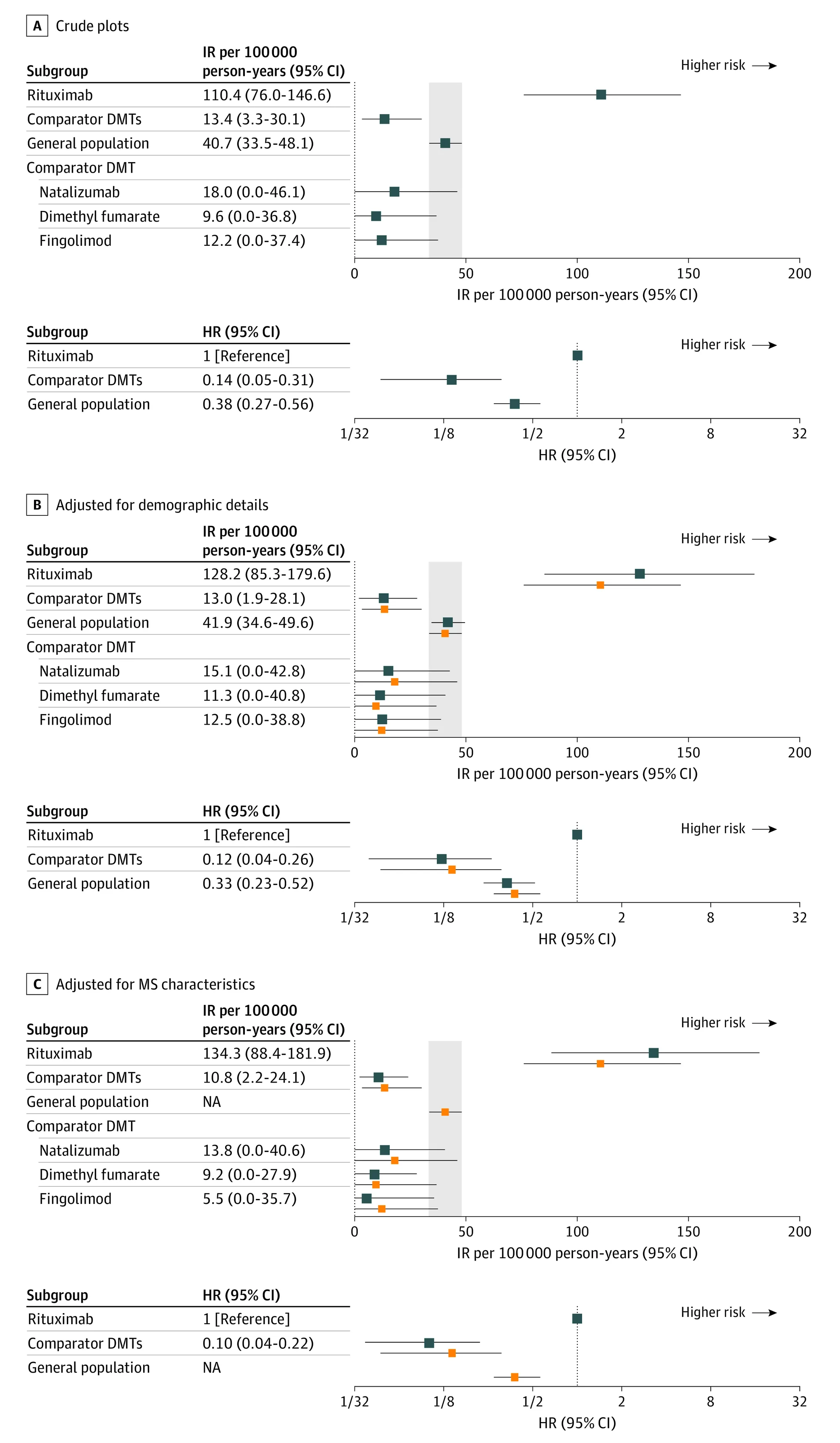
Forest Plots of Crude and Adjusted Incidence Rates (IRs) and Hazard Ratios (HRs) of Inflammatory Bowel Disease, Stratified by Exposure Group Penalized HRs with time since episode start as the time scale and rituximab as the reference. Comparator disease-modifying therapies (DMTs) grouped natalizumab, dimethyl fumarate, and fingolimod. For the adjusted IRs, separate models were fitted with the comparator DMTs grouped (upper rows) and ungrouped (lower rows). HRs were not estimated for the ungrouped therapies due to sparse data. The crude IR span for the general population is indicated in all IR plots, and the crude estimates are indicated in all adjusted plots (yellow markers). Demographics were adjusted for sex, age, country of birth (Sweden or other), civil status (married/partner), annual income, education, and area of residence in Sweden. Multiple sclerosis (MS) characteristics were adjusted for MS duration, year of therapy start, number of previous DMTs, number of previous relapses, and baseline Expanded Disability Status Scale, Symbol Digit Modalities Test, and Multiple Sclerosis Impact Scale.

### Survival Curves

IBD-free survival declined more steeply in the rituximab group than in the comparator DMT and general population groups, both before and after adjustment by weighting (Figure 2 and eFigure 4 in Supplement 1). However, cumulative incidence of IBD remained less than 2% in all groups 10 years after therapy start.

**Figure 2.**
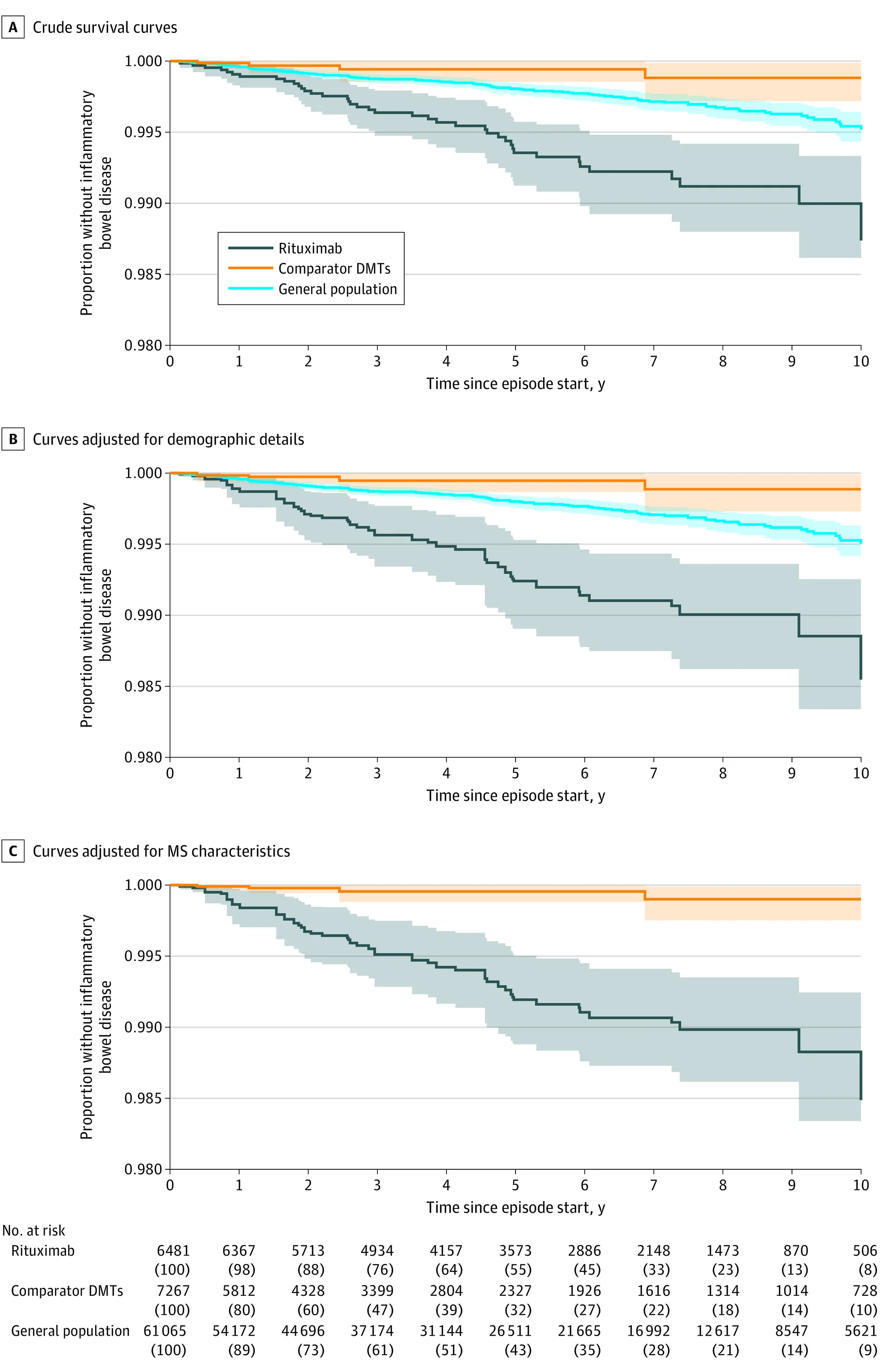
Crude and Adjusted Survival Curves for Time to Inflammatory Bowel Disease, Stratified by Exposure Group Estimated with Kaplan-Meier with pointwise CIs from bootstrapping. Numbers at risk stratified by group applies to all the plots shown. Comparator disease-modifying therapies (DMTs) grouped natalizumab, dimethyl fumarate, and fingolimod. Demographics were adjusted for sex, age, country of birth (Sweden or other), civil status (married/partner), annual income, education, and area of residence in Sweden. Multiple sclerosis (MS) characteristics were adjusted for MS duration, year of therapy start, number of previous DMTs, number of previous relapses, and baseline Expanded Disability Status Scale, Symbol Digit Modalities Test, and Multiple Sclerosis Impact Scale.

### HRs

Penalized crude Cox-estimated HRs for IBD were 0.14 (95% CI, 0.05-0.31) for the comparator DMTs and 0.38 (95% CI, 0.27-0.56) for the general population, with rituximab as the reference (Table 2 and Figure 1). After adjusting for demographics, the HR for the general population was 0.33 (95% CI, 0.23-0.52), and after additionally adjusting for MS characteristics, the HR for the comparator DMTs was 0.10 (95% CI, 0.04-0.22).

### Number Needed to Harm

The number needed to harm for rituximab compared with the comparator DMTs in the fully adjusted model was 131 (95% CI, 93-228) at 5 years, corresponding to 1 additional IBD case for every 131 individuals with MS treated with rituximab for 5 years instead of one of the comparator DMTs (Figure 3).

**Figure 3.**
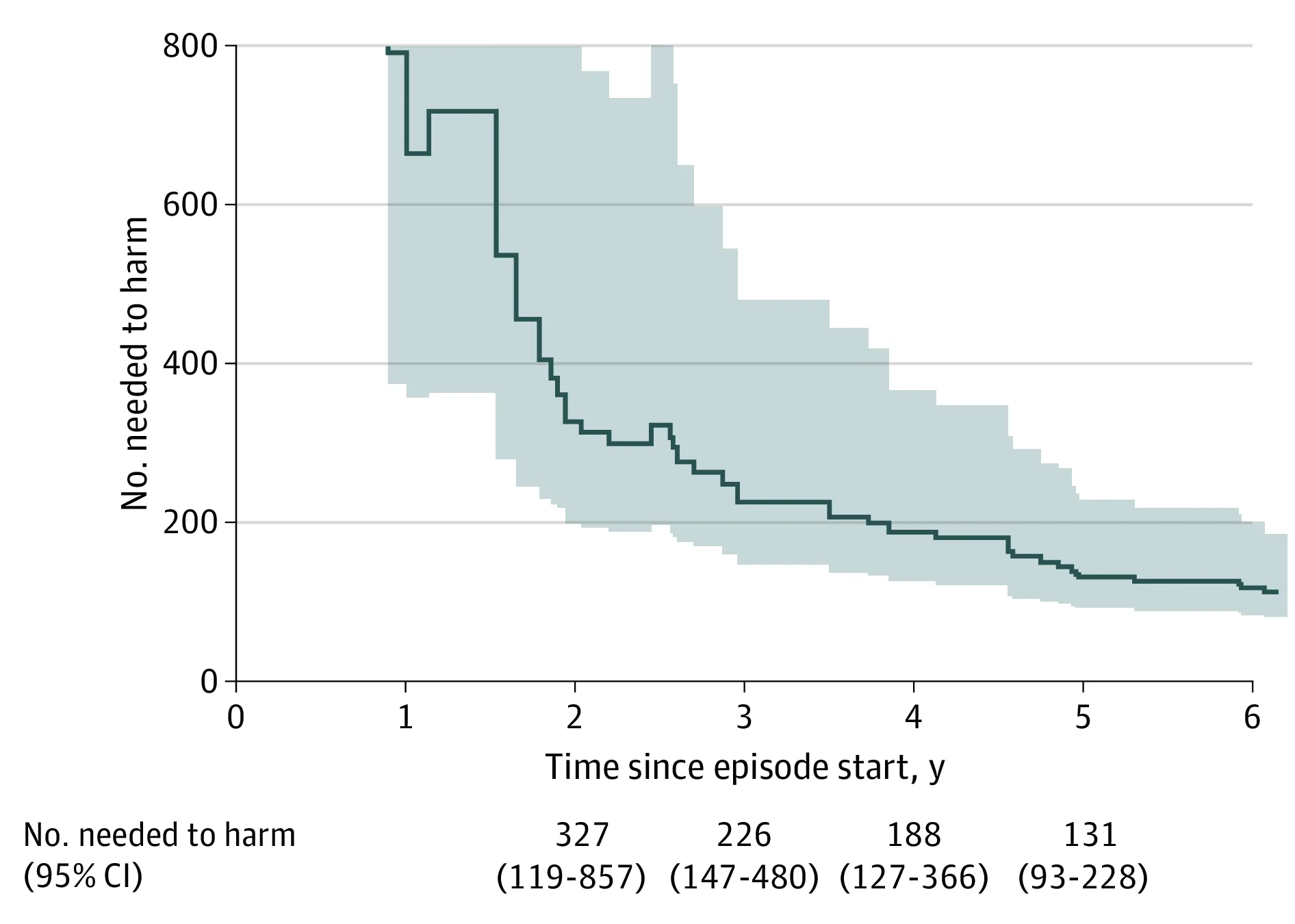
Time-Specific Numbers Needed to Harm With Rituximab vs the Comparator Disease-Modifying Therapies (DMTs) in the Fully Adjusted Model Comparator DMTs grouped natalizumab, dimethyl fumarate, and fingolimod and adjusted for sex, age, country of birth (Sweden or other), civil status (married/partner), annual income, education, area of residence in Sweden, multiple sclerosis duration, year of therapy start, number of previous DMTs, number of previous relapses, and baseline Expanded Disability Status Scale, Symbol Digit Modalities Test, and Multiple Sclerosis Impact Scale.

### Sensitivity Analysis

The sensitivity analyses supported the main results, with numbers needed to harm at 5 years of 158 (95% CI, 113-268) for an ever-treated approach to follow-up, 131 (95% CI, 86-237) using a stricter IBD definition, and 92 (95% CI, 60-196) when limiting the inclusion period to allow for higher overlap in start year between therapy groups. Detailed results from the sensitivity analyses can be found in eTables 6 to 8 and eFigures 5 to 20 in Supplement 1.

### Risk Factors for IBD

Comparing baseline characteristics stratified by both exposure and IBD status (eTable 4 in Supplement 1), rituximab episodes in which IBD developed were initiated at younger ages than those episodes that did not develop IBD, in individuals with a lower median disease duration. The difference in age was less apparent for the general population, and events were too few among the comparator DMTs for meaningful interpretation.

## Discussion

In this population-based cohort study of treatment episodes in individuals with RRMS, rituximab treatment was associated with an increased risk of IBD, compared with natalizumab, dimethyl fumarate, and fingolimod, corresponding to a number needed to harm of 131 at 5 years, after adjusting for differences in baseline characteristics.

To our knowledge, this was the first population-based study evaluating the risk of new-onset IBD during anti-CD20 treatment in RRMS. Previous epidemiological studies have demonstrated an association between MS and IBD, with an approximately 50% increased risk of either disease in the presence of the other,^31^ and white-matter lesions have been reported as a neurological manifestation of IBD.^32^

Recent case series^14^ have reported new-onset or exacerbated IBD during anti-CD20 treatment for MS, and an analysis of reports to the US Food and Drug Administration found that the anti-CD20 therapy ocrelizumab had a significantly higher reporting odds ratio of IBD compared with other MS DMTs.^19^ Cases of IBD have also been reported with rituximab treatment for other conditions, such as other autoimmune disorders and hematological malignancies (eMethods in Supplement 1).^21^

Our results expand on these reports to show that, among the therapies studied, only the anti-CD20 therapy rituximab was associated with an increased risk of IBD after adjustment for differences in characteristics at therapy start, primarily driven by Crohn disease. Our findings remained when maximizing follow-up time at the expense of specificity in outcome attribution, when using a stricter IBD definition, and when limiting the inclusion period to increase the overlap between the groups in sensitivity analyses, indicating that our results were robust to these assumptions.

Management of suspected anti-CD20–associated intestinal inflammation typically involves discontinuation of anti-CD20 therapy.^35^ Complete discontinuation may be safe for some individuals with long-term stable MS, but individual risk assessment is important, taking into consideration the person’s age, previous disease activity, and B-cell repopulation dynamics.^36^

### Strengths and Limitations

The main strengths of this study include the population-based design, high-quality national register data, an active-comparator design with confounder adjustment by inverse probability weighting, and a validated IBD definition. The use of national data allowed inclusion of a largely unselected population with RRMS, reducing selection bias and improving generalizability. Both the therapy data in the MS register and the diagnostic codes for IBD in the National Patient Register have previously been validated and found to be of high quality.^37^ Additionally, we used a previously validated IBD definition with a high positive predictive value.^25^ The use of the national registers also enabled adjustment for important potential confounders, including age, sex, socioeconomic factors, and, in the fully adjusted model, MS characteristics.

The main limitations of this study were the absence of other anti-CD20 therapies from the cohort, few IBD events among the comparator DMTs, potentially non–risk-neutral comparators, changing treatment patterns over time, missing data for MS characteristics, risk of residual confounding, the lack of clinical chart and pathology data, and the restriction to a Swedish population.

Due to the dominance of rituximab over other anti-CD20 therapies, such as ocrelizumab or ofatumumab, in Swedish MS care, we were not able to include other anti-CD20 drugs in the analysis. However, given that these therapies share the same mechanism of action, together with case reports of colitis for both ocrelizumab^14^ and ofatumumab,^20^ a drug-class effect is likely. In Sweden, individuals with MS tend to switch to rituximab and continue to receive it long term, resulting in shorter follow-up and fewer events for the comparator DMTs. We addressed this by grouping the comparator DMTs together for the main analyses, which allowed for more observed time and events and could be justified by their similar IBD incidence rates. We additionally added a 6-month extension to follow-up, used survival methods that accommodate differences in follow-up time, used penalization to reduce the risk of exaggerating the reported hazard ratios as a result of sparse data, and conducted a sensitivity analysis using an ever-treated approach to follow-up, which supported the main findings.

The risk of IBD with the comparator DMTs was lower than in the general population in our study, which appears to conflict with previous evidence that IBD risk is increased in individuals with MS.^31^ This could be an artifact of the low number of events, but it could also be explained by the other therapies possibly neutralizing this risk increase, as there is an overlap between some MS and IBD therapies. Fingolimod is a sphingosine-1-phosphate receptor modulator,^41^ the same class of immunomodulator as ozanimod, which is approved for the treatment of ulcerative colitis, albeit with a different receptor selectivity profile.^42^ Thus, it is possible that fingolimod may offer dual benefits for MS and IBD, but further studies are needed to confirm this. Natalizumab, which is an α4-integrin inhibitor, might have a similar dual effect on Crohn disease.^43^ Finally, there is preclinical evidence that dimethyl fumarate reduces inflammatory responses in experimental colitis mouse models.^44^ For our study, this means that the main comparators with rituximab were possibly not risk neutral, and the higher risk seen with rituximab could have been augmented through suppression of new-onset IBD in the comparator group or unmasking of prevalent IBD in the rituximab group for those previously treated with a comparator. However, it is unlikely that this would entirely explain our results as rituximab carried a greater excess risk relative to the general population (roughly 3-fold) than has been reported for MS alone (50%),^31^ and we found no evidence that previously masked disease inflated the number of rituximab cases through partial treatment with the comparators (eTable 5 in Supplement 1).

Changing treatment patterns for MS over time could have affected the results if there were also changes in diagnostic intensity or criteria for IBD during the same period. Treatment allocation was strongly associated with year of therapy start in our cohort, but limiting the analysis to an inclusion period with higher overlap between the groups did not alter our conclusions. Missing data in MS characteristics were addressed by multiple imputation and would only affect the fully adjusted models, which were in line with the models adjusted for demographic information. However, as in any observational study, residual confounding due to missing data or unmeasured factors, including established risk factors for IBD such as family history of IBD and smoking,^45^ cannot be excluded. These factors are unlikely to explain the entire association, however, as they are not expected to influence the choice of MS therapy.

The nationwide scope of our register linkage precluded access to clinical charts and pathology reports; IBD cases, therefore, could not be confirmed beyond *ICD* codes. To mitigate this limitation, we used an outcome definition previously validated against medical chart review (including pathology, endoscopy, and radiology reports), in which requiring at least 2 IBD diagnostic listings on separate dates yielded a positive predictive value of 93%.^25^ Additionally, as IBD typically follows a chronic course whereas drug-induced colitis is generally self-limited after withdrawal, we performed a sensitivity analysis using a stricter outcome definition requiring at least 3 IBD listings, with at least 1 year between the first and last, which yielded consistent results. Together with the long lookback period to exclude any prevalent IBD cases (based on both *ICD* and *ATC* codes), these measures reduce the risk of outcome misclassification, although residual misclassification cannot be excluded.

Finally, this study was conducted in Sweden, where 8% to 14% of the study population was born abroad, resulting in a relatively genetically homogeneous cohort, and further research is needed to confirm our findings in other populations.

## Conclusions

In this population-based cohort study of IBD risk in individuals with RRMS who are treated with DMT, the anti-CD20 therapy rituximab was associated with an increased risk of new-onset IBD after adjustment for differences in characteristics at therapy start, corresponding to a number needed to harm of 131 at 5 years, compared with natalizumab, dimethyl fumarate, and fingolimod. Clinicians and individuals with MS should be aware of the risk of IBD and remain vigilant for gastrointestinal symptoms during treatment with anti-CD20 therapy. Careful consideration should also be given before initiating treatment with anti-CD20 therapy in those with MS and a history of IBD. Further research is needed to explore whether treatment modifications or monitoring strategies could mitigate IBD risk with anti-CD20 therapy and to establish clinical risk factors for anti-CD20–associated IBD.

## Notes

Supplement 1.
eMethods.
**eTable 1.** Inclusion in Treated Population **eTable 2.** Inclusion in General Population **eTable 3.** Missing Data **eTable 4.** Baseline Characteristics Stratified by Exposure and Outcome Status **eTable 5.** IBD Cases in the Rituximab Group Stratified by Previous Comparator Treatment **eFigure 1.** Distribution of Follow-Up Time Stratified by Exposure **eFigure 2.** Distribution of Time to Outcome, Stratified by Exposure **eFigure 3.** Distribution of Time to Therapy Stop for IBD Cases in the Rituximab Group **eFigure 4.** Crude Survival Curves for Time to IBD, Stratified by Exposure Group **eTable 6.** Observed Time, Events, and Incidence Rates, Stratified by Exposure Group **eFigure 5.** Distribution of Follow-Up Time Stratified by Exposure **eFigure 6.** Distribution of Time to Outcome, Stratified by Exposure **eFigure 7.** Crude and Adjusted Survival Curves for Time to Inflammatory Bowel Disease **eFigure 8.** Forest Plots of Incidence Rates and Hazard Ratios of Inflammatory Bowel Disease **eFigure 9.** Numbers Needed to Harm With Rituximab vs the Comparator DMTs **eTable 7.** Observed Time, Events, and Incidence Rates, Stratified by Exposure Group **eFigure 10.** Distribution of Follow-Up Time Stratified by Exposure **eFigure 11.** Distribution of Time to Outcome, Stratified by Exposure **eFigure 12.** Crude and Adjusted Survival Curves for Time to Inflammatory Bowel Disease **eFigure 13.** Forest Plots of Incidence Rates and Hazard Ratios of Inflammatory Bowel Disease **eFigure 14.** Numbers Needed to Harm With Rituximab vs the Comparator DMTs **eFigure 15.** Distributions of Index (Episode Start) Year Stratified by Exposure Group **eTable 8.** Observed Time, Events, and Incidence Rates, Stratified by Exposure Group **eFigure 16.** Distribution of Follow-Up Time Stratified by Exposure **eFigure 17.** Distribution of Time to Outcome, Stratified by Exposure **eFigure 18.** Crude and Adjusted Survival Curves for Time to Inflammatory Bowel Disease **eFigure 19.** Forest Plots of Incidence Rates and Hazard Ratios of Inflammatory Bowel Disease **eFigure 20.** Numbers Needed to Harm With Rituximab vs the Comparator DMTs

Supplement 2.
Data Sharing Statement.
