# Supplementary material for: Risk of Inflammatory Bowel Disease After Anti-CD20 Treatment in Multiple Sclerosis
Source: JAMA Neurol. 2026 Oct 22:e263439. Online ahead of print. doi: 10.1001/jamaneurol.2026.3439 (PMC13691918; doi:10.1001/jamaneurol.2026.3439)
Supplement: Supplementary file 1 [file jamaneurol-e263439-s001.pdf]

Supplementary Online Content

Kallin S, Svenningsson A, Olén O, Hedin C, Lycke J, Alping P. Risk of inflammatory bowel disease after anti-CD20 treatment in multiple sclerosis. *JAMA Neurol*. Published online October 22, 2026. doi:10.1001/jamaneurol.2026.3439

eMethods.

eTable 1. Inclusion in Treated Population

eTable 2. Inclusion in General Population

eTable 3. Missing Data

eTable 4. Baseline Characteristics Stratified by Exposure and Outcome Status

eTable 5. IBD Cases in the Rituximab Group Stratified by Previous Comparator Treatment

eFigure 1. Distribution of Follow-Up Time Stratified by Exposure

eFigure 2. Distribution of Time to Outcome, Stratified by Exposure

eFigure 3. Distribution of Time to Therapy Stop for IBD Cases in the Rituximab Group

eFigure 4. Crude Survival Curves for Time to IBD, Stratified by Exposure Group

eTable 6. Observed Time, Events, and Incidence Rates, Stratified by Exposure Group

eFigure 5. Distribution of Follow-Up Time Stratified by Exposure

eFigure 6. Distribution of Time to Outcome, Stratified by Exposure

eFigure 7. Crude and Adjusted Survival Curves for Time to Inflammatory Bowel Disease

eFigure 8. Forest Plots of Incidence Rates and Hazard Ratios of Inflammatory Bowel Disease

eFigure 9. Numbers Needed to Harm With Rituximab vs the Comparator DMTs

eTable 7. Observed Time, Events, and Incidence Rates, Stratified by Exposure Group

eFigure 10. Distribution of Follow-Up Time Stratified by Exposure

eFigure 11. Distribution of Time to Outcome, Stratified by Exposure

eFigure 12. Crude and Adjusted Survival Curves for Time to Inflammatory Bowel Disease

© 2026 Kallin S et al. *JAMA Neurology*.

eFigure 13. Forest Plots of Incidence Rates and Hazard Ratios of Inflammatory Bowel Disease

eFigure 14. Numbers Needed to Harm With Rituximab vs the Comparator DMTs

eFigure 15. Distributions of Index (Episode Start) Year Stratified by Exposure Group

eTable 8. Observed Time, Events, and Incidence Rates, Stratified by Exposure Group

eFigure 16. Distribution of Follow-Up Time Stratified by Exposure

eFigure 17. Distribution of Time to Outcome, Stratified by Exposure

eFigure 18. Crude and Adjusted Survival Curves for Time to Inflammatory Bowel Disease

eFigure 19. Forest Plots of Incidence Rates and Hazard Ratios of Inflammatory Bowel Disease

eFigure 20. Numbers Needed to Harm With Rituximab vs the Comparator DMTs

This supplementary material has been provided by the authors to give readers additional information about their work.

eMethods.

Setting and data sources

In this population-based cohort study of IBD risk with MS treatment, we linked the Swedish MS register[1] with national demographic and medical registers using the personal identity number unique to each resident in Sweden. Data cut-off was 2025-12-31.

Sweden has tax-funded universal healthcare and persons with MS are primarily treated at public neurology clinics where the Swedish MS register is used as a clinical documentation tool. The information in the register includes MS characteristics, clinical course, investigations, and treatment. Although participation in the register is voluntary, all specialist neurology clinics in Sweden are represented and the estimated completeness is 85%.[2,3]

The National Patient Register contains dates and diagnosis codes (International Classification of Diseases [ICD-10]) for all inpatient care since 1987, and secondary care visits to a physician since 2001.[4] The Prescribed Drug Register contains dates and drug codes (Anatomical Therapeutic Chemical [ATC]) for all medications dispensed from pharmacies since 2005.[5]

The national demographic registers hold data on age, sex, birth region, and death (the total population register), education (the Swedish Longitudinal Database for Health Insurance and Labour Market Studies), income (the taxation register), and migration (the migration register) for all residents in Sweden.

Individuals were included in the register linkage as index persons if they were registered in the MS register, had a diagnosis code in inpatient or secondary outpatient care indicative of MS, and/or had collected an MS-specific therapy from a pharmacy. Of these, only persons with at least one of the studied therapies registered in the Swedish MS register were included in our study. Each index person in the linkage was matched to five comparators from the general Swedish population (i.e. not in the index population at the time) on age, sex, and residential area at the index date (date of diagnosis in MS register, first MS diagnosis code in the National Patient Register, or first DMT dispensation in the Prescribed Drug Register). From this comparator population, we then created "general population episodes" for each therapy episode included in our study, maintaining the original matching. Example: Five persons in the general population were matched to one person with MS in the Swedish MS register and for each therapy episode this index person had, the equivalent general population episodes were created for the general population comparators (using the same start and stop dates as for the index person’s therapy episode). The clustering on index persons was taken into account using cluster bootstrap to preserve valid confidence intervals.

Study population and prior IBD suspicion

To only capture new-onset IBD as the study outcome, exposure episodes were excluded if there was any suspicion of IBD prior to the index date, based on inpatient and secondary outpatient care visits (ICD-10 codes), as well as prescribed drugs collected from pharmacies (ATC codes). All exposure episodes had at least ten years of lookback time before the index date for inpatient/outpatient visits and five years for prescribed drugs, with any observation of the specified codes pre-treatment resulting in exclusion. Corticosteroids were not included as they are not specific for IBD. Biologic drugs without the option of subcutaneous administration (i.e. only infusion) were not included as they are not collected from pharmacies.

ICD-10

- **K50:** Crohn's disease
- **K51:** Ulcerative colitis
- **K52.3:** Indeterminate colitis
- **K52.8:** Other specified noninfective gastroenteritis and colitis
- **K52.9:** Noninfective gastroenteritis and colitis, unspecified

© 2026 Kallin S et al. *JAMA Neurology*.

ATC

- **A07EC01:** sulfasalazine
- **A07EC02:** mesalazine
- **A07EC03:** olsalazine
- **A07EC04:** balsalazide
- **L04AX01:** azathioprine
- **L01BB02:** mercaptopurine
- **L04AX03:** methotrexate
- **L04AE02:** ozanimod
- **L04AE05:** etrasimod
- **L04AF01:** tofacitinib
- **L04AF04:** filgotinib
- **L04AB02:** infliximab
- **L04AB04:** adalimumab
- **L04AB06:** golimumab
- **L04AG05:** vedolizumab
- **L04AC05:** ustekinumab

Outcome definition

The outcome of this study was new-onset IBD after episode start, identified through ICD-10 codes from the National Patient Register in accordance with a previously validated definition:[6] At least two observations on separate dates in inpatient or secondary outpatient care, with the code **K50** (Crohn's disease), **K51** (ulcerative colitis), and/or **K52.3** (indeterminate colitis). The date of the first code was used as the outcome date; therefore, only this code had to be within the follow-up period. We further classified the IBD outcome, based on the ICD codes registered for the first two observations, into:[7] **Crohn's disease** (only K50 codes), **ulcerative colitis** (only K51 codes), or **unclassified IBD** (any other combination of K50, K51, and K52.3). The study population was selected as those without prior IBD suspicion (see above), and with at least ten years of register data before episode start, to make misclassification of the outcome due to prevalent IBD unlikely.

Our outcome definition was based on a validation of IBD in the National Patient Register through medical chart review by Jakobsson et al., where a case definition requiring at least two recorded IBD diagnoses (from hospitalizations or non-primary outpatient care) corresponded to a positive predictive value of 93% (95% CI: 87–97%) for any IBD, 79% (66–88%) for ulcerative colitis, and 72% (60–82%) for Crohn's disease. The lower values for the individual subtypes largely reflected diagnostic reclassification between UC and CD rather than misclassification as non-IBD. A subset analysis of 17 excluded patients with only a single recorded diagnosis supported the definition, as eight of them did not meet any criteria for IBD, supporting that the two-diagnosis requirement was effective at filtering out false-positive cases.

The high positive predictive value of this validated IBD definition, together with the long lookback time before episode start and exclusion of any suspected IBD cases, substantially reduces the risk of misclassification of the outcome in our study.

Suggested mechanisms for IBD with anti-CD20 treatment

Experimental models suggest that anti-CD20 treatment may disrupt gastrointestinal immune homeostasis. In rituximab-treated mice, intestinal tissue showed increased proinflammatory cytokines (TNFα, IL-1β, IL-6) and reduced IL-10, alongside expansion of Th1 and Th17 subsets in the mesenteric lymph nodes.[8] B cells, which are targeted by anti-CD20 therapy, also appear to have a role in suppressing pathogenic T helper cells in colitis models.[9] Apart from IBD, other autoimmune diseases are also more common in MS compared with the general population, including autoimmune thyroiditis, type 1 diabetes mellitus, and psoriasis.[10,11] However, there have been no indications that

© 2026 Kallin S et al. *JAMA Neurology*.

anti-CD20 therapy increases the risk of these conditions, with the possible exception of psoriasis,[12] suggesting that the IBD association may reflect gut-specific immune disruption rather than a general increase in autoimmune risk. Quesada-Simó et al. recently proposed diagnostic criteria for anti-CD20-induced IBD.[13] However, histological findings are highly variable, ranging from IBD-like patterns to microscopic colitis and apoptotic colonopathy, making it unclear whether anti-CD20-associated colitis actually represents a distinct entity or instead reflects a combination of new-onset IBD in susceptible individuals and direct mucosal effects of the treatment.[14,15]

Sparse data and penalization

There were few outcome events in the non-rituximab therapy groups in our study, which is in line with the hypothesis that rituximab is increasing the risk of IBD. However, the low number of events can cause statistical issues, as sparse data risk pushing estimates toward more extreme values. To mitigate this, we grouped the comparator DMTs together for our main results, to make the comparisons with rituximab more reliable. We also used penalization in the Cox regression as recommended by Greenland et al. for sparse data.[16] We introduced penalization through ridge regression, choosing the penalization strength based on a prior assumption of the range of plausible values for the hazard ratios relative to rituximab. We chose a wide symmetric (on the HR scale), normally distributed prior with 95% of its probability mass between 1/40 and 40, corresponding to a lambda entering the ridge regression penalization term ($\frac{\lambda}{2}\|\beta\|_2^2$) of $\lambda = \left(\frac{1.96}{\ln(40)}\right)^2 = 0.2823$ ....

Missing data

Missing data were addressed by stochastic predictive mean matching imputation (20 burn-in iterations), including all variables from the weighting model, as well as an indicator of the outcome and the Nelson-Aalen estimate of the cumulative hazard within each exposure group. To reduce complexity, the few observations with missing data among the demographic variables were excluded when these variables were used for adjustment and imputation was performed only for MS characteristics. For point estimates, the stochastic imputation was performed 50 times (i.e. multiple imputation), with the estimation of weights and model fitting done separately for each imputed dataset and summarized as the mean. The bootstrapped confidence intervals used ten stochastic imputations within each bootstrap and averaged the weights over the imputed datasets before fitting the weighted models, giving one estimate per model and bootstrap for use with the percentile method.[17-19]

Software

Data management and analysis were performed using Python 3.14 and R 4.6.0. Data management was performed with Polars (Python), imputation with MICE (R), Kaplan-Meier with lifelines (Python), and multinomial logistic regression with scikit-learn (Python). Penalized Cox regression was performed using a custom model purpose-built with JAX (Python).

References (supplement)

1. Swedish Neuro Registries. Multiple Sclerosis. https://www.neuroreg.se/en/multipel-skleros/.
2. Alping P, Piehl F, Langer-Gould A, Frisell T, Group C-MS. Validation of the Swedish Multiple Sclerosis Register: Further Improving a Resource for Pharmacoepidemiologic Evaluations. *Epidemiology.* 2019;30(2):230-233.
3. Alping P, Frisell T, He A, Hillert J, Fink K, McKay KA. Completeness and generalizability of the Swedish MS register. *Eur J Epidemiol.* 2025;40(8):905-913.
4. Everhov AH, Frisell T, Osooli M, et al. Diagnostic accuracy in the Swedish national patient register: a review including diagnoses in the outpatient register. *Eur J Epidemiol.* 2025;40(3):359-369.
5. Wettermark B, Hammar N, Fored CM, et al. The new Swedish Prescribed Drug Register--opportunities for pharmacoepidemiological research and experience from the first six months. *Pharmacoepidemiol Drug Saf.* 2007;16(7):726-735.
6. Jakobsson GL, Sternegard E, Olen O, et al. Validating inflammatory bowel disease (IBD) in the Swedish National Patient Register and the Swedish Quality Register for IBD (SWIBREG). *Scand J Gastroenterol.* 2017;52(2):216-221.

© 2026 Kallin S et al. *JAMA Neurology*.

7. Everhov AH, Sachs MC, Malmborg P, et al. Changes in inflammatory bowel disease subtype during follow-up and over time in 44,302 patients. *Scand J Gastroenterol.* 2019;54(1):55-63.
8. Zhao B, Zhou B, Dong C, et al. Lactobacillus reuteri Alleviates Gastrointestinal Toxicity of Rituximab by Regulating the Proinflammatory T Cells in vivo. *Front Microbiol.* 2021;12:645500.
9. Mizoguchi E, Mizoguchi A, Preffer FI, Bhan AK. Regulatory role of mature B cells in a murine model of inflammatory bowel disease. *Int Immunol.* 2000;12(5):597-605.
10. Barkhane Z, Elmadi J, Satish Kumar L, Pugalenthi LS, Ahmad M, Reddy S. Multiple Sclerosis and Autoimmunity: A Veiled Relationship. *Cureus.* 2022;14(4):e24294.
11. Aslan D, Bourabia S, Kowall B, et al. The impact of autoimmune comorbidities on multiple sclerosis progression: insights from a longitudinal single-center study. *J Neurol.* 2025;272(9):607.
12. Molazadeh N, Ala S, Karaminia M, Sahraian MA. Rituximab induced psoriasis in a patient with multiple sclerosis: A case report and literature review. *Neuroimmunology Reports.* 2021;1:100027.
13. Quesada-Simo A, Garrido-Marin A, Nos P, Gil-Perotin S. Impact of Anti-CD20 therapies on the immune homeostasis of gastrointestinal mucosa and their relationship with de novo intestinal bowel disease in multiple sclerosis: a review. *Front Pharmacol.* 2023;14:1186016.
14. Eckmann JD, Chedid V, Quinn KP, Bonthu N, Nehra V, Raffals LE. De Novo Colitis Associated With Rituximab in 21 Patients at a Tertiary Center. *Clin Gastroenterol Hepatol.* 2020;18(1):252-253.
15. Challa B, Esnakula AK. Histologic manifestations of ocrelizumab-associated intestinal and hepatic injury in patients with multiple sclerosis. *Histopathology.* 2024;84(5):765-775.
16. Greenland S, Mansournia MA, Altman DG. Sparse data bias: a problem hiding in plain sight. *BMJ.* 2016;352:i1981.
17. Schomaker M, Heumann C. Bootstrap inference when using multiple imputation. *Stat Med.* 2018;37(14):2252-2266.
18. Nguyen TQ, Stuart EA. Multiple imputation for propensity score analysis with covariates missing at random: some clarity on "within" and "across" methods. *Am J Epidemiol.* 2024;193(10):1470-1476.
19. Li CX, Zivich PN. Invited commentary: mixing multiple imputation and bootstrapping for variance estimation. *Am J Epidemiol.* 2024;193(10):1477-1481.

© 2026 Kallin S et al. *JAMA Neurology*.

eTable 1: Inclusion in treated population

Criteria	Rituximab	Natalizumab	Dimethyl Fumarate	Fingolimod
All therapies	11,078 (100%)	5,569 (100%)	3,707 (100%)	1,971 (100%)
In inclusion period	10,278 (93%)	3,869 (69%)	3,681 (99%)	1,947 (99%)
RRMS at therapy start	8,378 (76%)	3,659 (66%)	3,496 (94%)	1,824 (93%)
In age interval	7,454 (67%)	3,439 (62%)	3,142 (85%)	1,716 (87%)
≥10 years lookback	6,757 (61%)	3,093 (56%)	2,861 (77%)	1,585 (80%)
No previous IBD	6,481 (59%)	2,976 (53%)	2,767 (75%)	1,524 (77%)

RRMS=Relapsing-Remitting Multiple Sclerosis; IBD=Inflammatory Bowel Disease

eTable 2: Inclusion in general population

Criteria	Rituximab	Natalizumab	Dimethyl Fumarate	Fingolimod
All matched	32,681 (100%) [5.0]	15,004 (100%) [5.0]	13,926 (100%) [5.0]	7,699 (100%) [5.1]
Not MS before index date	32,644 (100%) [5.0]	14,990 (100%) [5.0]	13,907 (100%) [5.0]	7,684 (100%) [5.0]
In age interval	32,521 (100%) [5.0]	14,958 (100%) [5.0]	13,860 (100%) [5.0]	7,670 (100%) [5.0]
≥10 years lookback	29,814 (91%) [4.6]	13,467 (90%) [4.5]	12,739 (91%) [4.6]	7,211 (94%) [4.7]
No previous IBD	28,752 (88%) [4.4]	13,027 (87%) [4.4]	12,288 (88%) [4.4]	6,998 (91%) [4.6]

Numbers within brackets represent the mean number of matched general population episodes per treatment episode.
RRMS=Relapsing-Remitting Multiple Sclerosis; IBD=Inflammatory Bowel Disease

eTable 3: Missing data

Variable	Rituximab	Natalizumab	Dimethyl Fumarate	Fingolimod	General Population
N	6,481	2,976	2,767	1,524	61,065
Born in Sweden	0 (0.0)	0 (0.0)	1 (0.0)	1 (0.1)	6 (0.0)
Married/Partner	2 (0.0)	1 (0.0)	1 (0.0)	1 (0.1)	760 (1.2)
Yearly income (thousand SEK)	2 (0.0)	1 (0.0)	1 (0.0)	1 (0.1)	475 (0.8)
Education	22 (0.3)	11 (0.4)	6 (0.2)	5 (0.3)	490 (0.8)
EDSS	3,490 (53.8)	1,824 (61.3)	1,489 (53.8)	794 (52.1)	-
SDMT	3,725 (57.5)	1,367 (45.9)	1,444 (52.2)	540 (35.4)	-
MSIS-29 Physical	3,610 (55.7)	1,445 (48.6)	1,403 (50.7)	527 (34.6)	-
MSIS-29 Psychological	3,608 (55.7)	1,445 (48.6)	1,403 (50.7)	527 (34.6)	-
Area in Sweden	2 (0.0)	1 (0.0)	1 (0.0)	1 (0.1)	760 (1.2)

Only variables with any missing data are shown. SEK=Swedish krona; EDSS=Expanded Disability Status Scale; SDMT=Symbol Digit Modalities Test; MSIS-29=Multiple Sclerosis Impact Scale

© 2026 Kallin S et al. *JAMA Neurology*.

eTable 4: Baseline characteristics stratified by exposure and outcome status

Variable	Rituximab - No IBD	Rituximab - IBD	Comparator DMTs - No IBD	Comparator DMTs - IBD	General Population - No IBD	General Population - IBD
N	6,441	40	7,263	4	60,945	120
Year of episode start	2018 (2016–2021)	2016 (2015–2018)	2015 (2014–2018)	2014.5 (2014–2015)	2017 (2014–2020)	2016 (2014–2018)
Age, years	38.4 (9.1)	35.6 (9.3)	36.8 (8.8)	33.7 (11.0)	37.8 (9.0)	36.4 (9.8)
Sex, female	4,518 (70.1)	29 (72.5)	5,315 (73.2)	3 (75.0)	43,726 (71.7)	75 (62.5)
Born in Sweden	5,777 (89.7)	35 (87.5)	6,640 (91.4)	3 (75.0)	52,532 (86.2)	104 (86.7)
Married/Partner	2,354 (36.6)	12 (30.0)	2,750 (37.9)	0 (0.0)	22,172 (36.8)	35 (29.2)
Yearly income (thousand SEK)	317.1 (123.6–470.1)	236.3 (46.9–402.4)	285.9 (117.1–429.2)	284.3 (244.7–323.9)	328.8 (142.2–464.7)	245.6 (89.6–415.6)
Education, primary	516 (8.0)	4 (10.0)	596 (8.2)	0 (0.0)	5,263 (8.7)	12 (10.0)
Education, secondary	2,873 (44.8)	15 (37.5)	3,307 (45.7)	3 (75.0)	26,729 (44.2)	63 (52.5)
Education, higher	3,030 (47.2)	21 (52.5)	3,338 (46.1)	1 (25.0)	28,463 (47.1)	45 (37.5)
Years since MS onset	4.9 (1.1–11.4)	4.1 (1.7–13.2)	4.5 (1.0–10.2)	5.7 (2.7–8.7)	-	-
No. of prev. DMTs	1.0 (0.0–2.0)	1.0 (0.0–2.0)	1.0 (0.0–2.0)	1.0 (1.0–1.0)	-	-
Previous Natalizumab	1,929 (29.9)	9 (22.5)	1,175 (16.2)	0 (0.0)	-	-
Previous Fingolimod	702 (10.9)	1 (2.5)	477 (6.6)	0 (0.0)	-	-
Previous Rituximab	150 (2.3)	0 (0.0)	260 (3.6)	0 (0.0)	-	-
Previous Dimethyl Fumarate	1,154 (17.9)	7 (17.5)	699 (9.6)	1 (25.0)	-	-
No. of prev. relapses	1.0 (0.0–2.0)	1.0 (0.0–3.0)	1.0 (0.0–2.0)	1.5 (1.0–2.0)	-	-
EDSS, mild	2,344 (78.9)	18 (81.8)	2,515 (79.6)	1 (100.0)	-	-
EDSS, moderate	526 (17.7)	4 (18.2)	527 (16.7)	0 (0.0)	-	-
EDSS, severe	99 (3.3)	0 (0.0)	117 (3.7)	0 (0.0)	-	-
SDMT	53.0 (46.0–61.0)	53.0 (46.0–60.0)	53.0 (46.0–60.0)	45.0 (45.0–47.0)	-	-
MSIS-29 Physical	1.5 (1.1–2.2)	1.6 (1.1–2.2)	1.5 (1.1–2.2)	1.4 (1.2–1.5)	-	-
MSIS-29 Psychological	2.1 (1.4–2.9)	2.7 (1.4–3.2)	2.0 (1.4–2.9)	3.2 (3.1–3.2)	-	-
Area, Stockholm	1,781 (27.7)	10 (25.0)	1,183 (16.3)	0 (0.0)	12,958 (21.5)	35 (29.2)
Area, East Middle Sweden	1,095 (17.0)	9 (22.5)	1,084 (14.9)	0 (0.0)	9,640 (16.0)	18 (15.0)
Area, South Sweden	780 (12.1)	4 (10.0)	1,351 (18.6)	0 (0.0)	9,069 (15.1)	11 (9.2)
Area, North Middle Sweden	595 (9.2)	3 (7.5)	580 (8.0)	1 (25.0)	5,201 (8.6)	11 (9.2)
Area, Middle Norrland	296 (4.6)	0 (0.0)	218 (3.0)	0 (0.0)	2,323 (3.9)	2 (1.7)
Area, Upper Norrland	459 (7.1)	1 (2.5)	230 (3.2)	0 (0.0)	3,043 (5.1)	4 (3.3)
Area, Småland and the islands	400 (6.2)	7 (17.5)	599 (8.3)	1 (25.0)	4,595 (7.6)	6 (5.0)
Area, West Sweden	1,033 (16.0)	6 (15.0)	2,015 (27.8)	2 (50.0)	13,356 (22.2)	33 (27.5)
Crohn's Disease	-	25 (62.5)	-	1 (25.0)	-	35 (29.2)
Ulcerative Colitis	-	6 (15.0)	-	3 (75.0)	-	67 (55.8)
Unclassified IBD	-	9 (22.5)	-	0 (0.0)	-	18 (15.0)

Mean (standard deviation), median (interquartile range), or number (proportion). Missing values excluded. IBD=Inflammatory Bowel Disease; SEK=Swedish krona; EDSS=Expanded Disability Status Scale; MSIS-29=Multiple Sclerosis Impact Scale; SDMT=Symbol Digit Modalities Test

© 2026 Kallin S et al. *JAMA Neurology*.

eTable 5: IBD cases in the rituximab group stratified by previous comparator treatment

Previous treatment	N	Person-years (median)	Events (proportion)	Incidence Rate
No previous comparator	3,305	19,023 (5.7)	25 (0.76%)	131.4
Natalizumab	1,411	7,345 (4.4)	7 (0.50%)	95.3
Dimethyl Fumarate	895	4,585 (5.3)	6 (0.67%)	130.8
Fingolimod	274	1,858 (7.2)	0 (0.00%)	0.0
Combination	596	3,405 (5.7)	2 (0.34%)	58.7

Incidence rates (IR) per 100,000 person-years. Combination=Previous treatment with more than one comparator DMT. IBD=Inflammatory Bowel Disease

© 2026 Kallin S et al. *JAMA Neurology*.

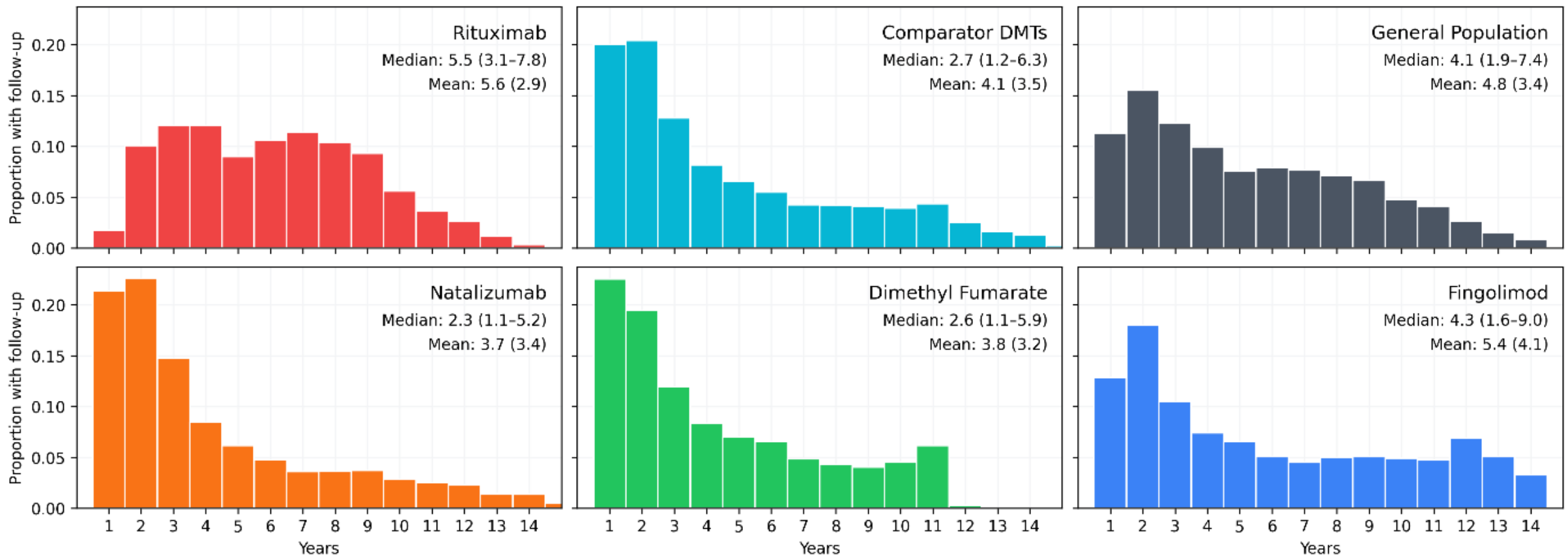

eFigure 1: Distribution of follow-up time stratified by exposure
Comparator disease-modifying therapies (DMTs) grouped natalizumab, dimethyl fumarate, and fingolimod.

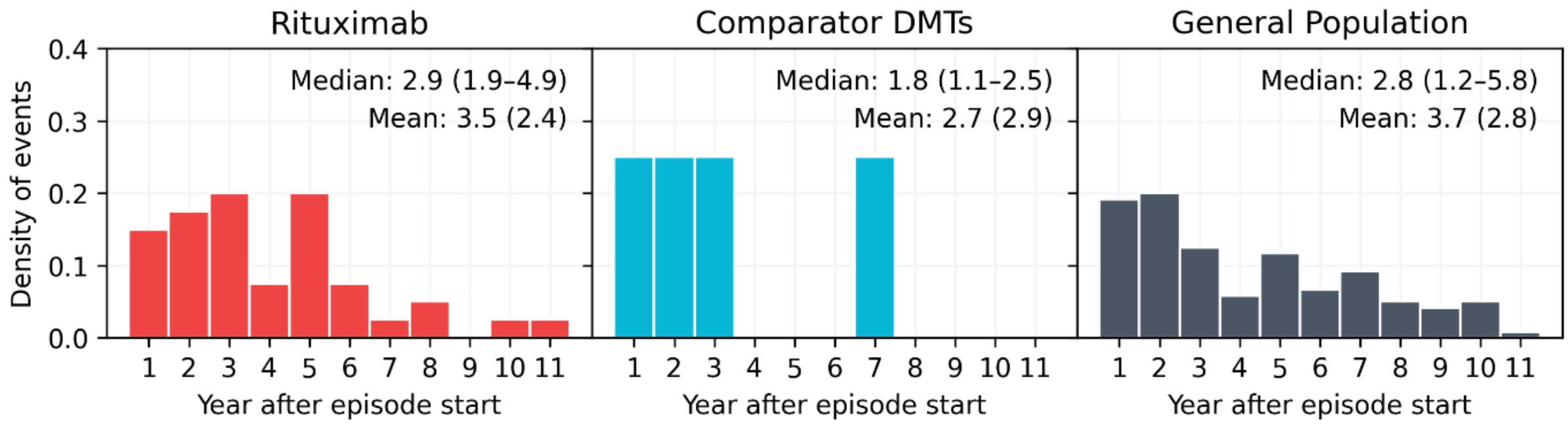

eFigure 2: Distribution of time to outcome, stratified by exposure
Comparator disease-modifying therapies (DMTs) grouped natalizumab, dimethyl fumarate, and fingolimod.

© 2026 Kallin S et al. *JAMA Neurology*.

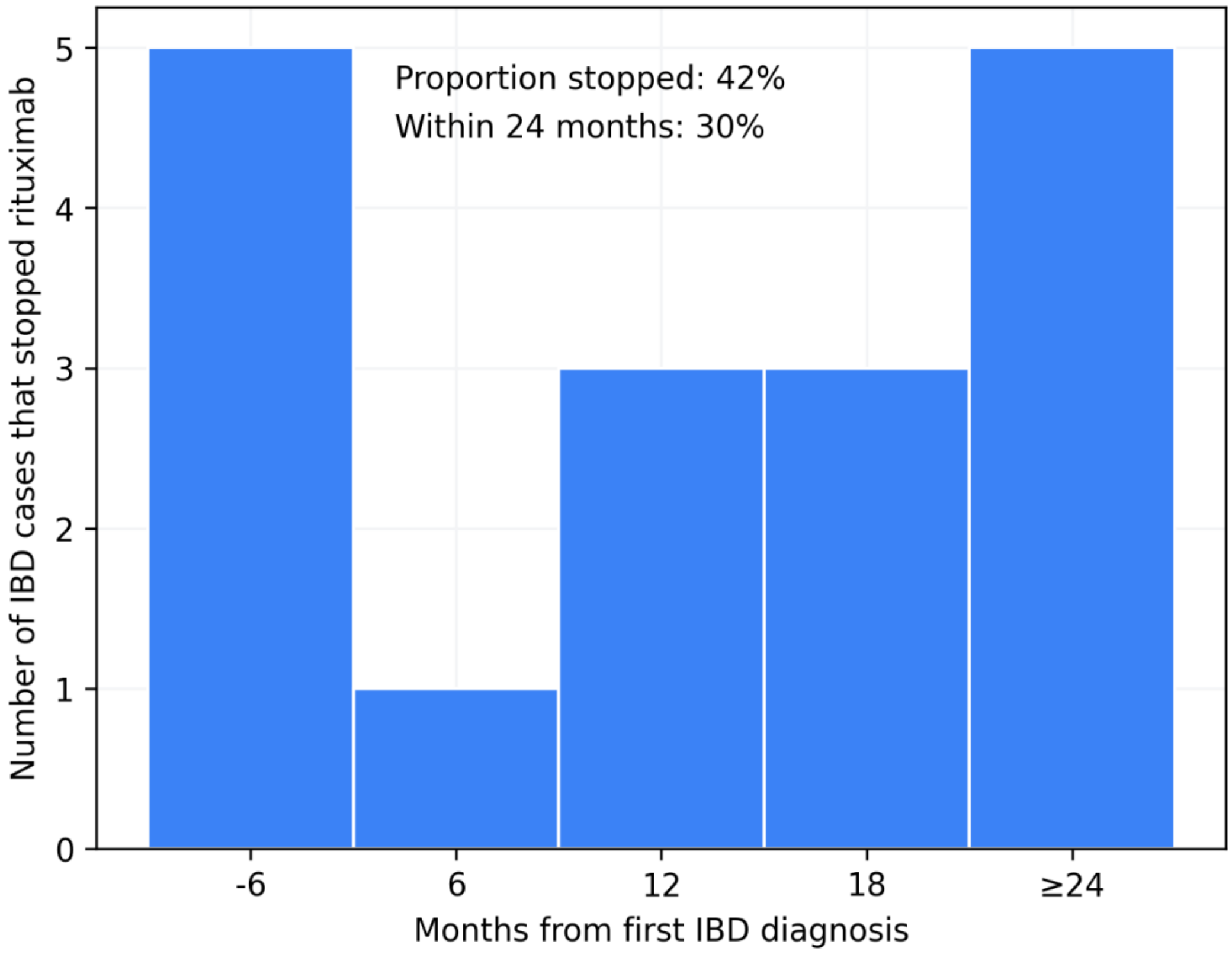

eFigure 3: Distribution of time to therapy stop for IBD cases in the rituximab group
Distribution shown for those who stopped rituximab, with proportion that stopped indicated.
IBD=Inflammatory Bowel Disease

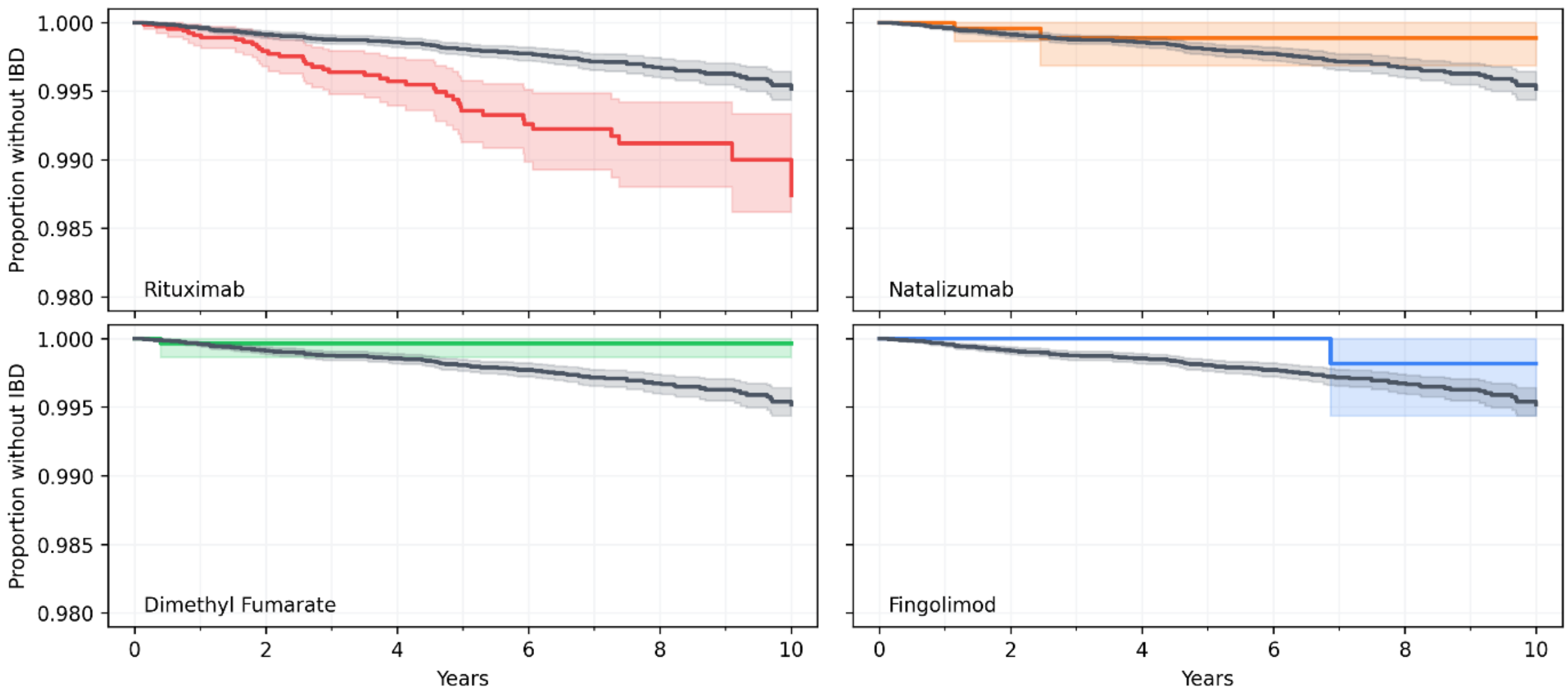

Years since start	0	1	2	3	4	5	6	7	8	9	10
Rituximab	6,481 (100)	6,367 (98)	5,713 (88)	4,934 (76)	4,157 (64)	3,573 (55)	2,886 (45)	2,148 (33)	1,473 (23)	870 (13)	506 (8)
Natalizumab	2,976 (100)	2,340 (79)	1,668 (56)	1,230 (41)	978 (33)	795 (27)	653 (22)	546 (18)	438 (15)	328 (11)	243 (8)
Dimethyl Fumarate	2,767 (100)	2,144 (77)	1,606 (58)	1,275 (46)	1,045 (38)	851 (31)	669 (24)	535 (19)	417 (15)	304 (11)	178 (6)
Fingolimod	1,524 (100)	1,328 (87)	1,054 (69)	894 (59)	781 (51)	681 (45)	604 (40)	535 (35)	459 (30)	382 (25)	307 (20)
General Population	61,065 (100)	54,172 (89)	44,696 (73)	37,174 (61)	31,144 (51)	26,511 (43)	21,665 (35)	16,992 (28)	12,617 (21)	8,547 (14)	5,621 (9)

eFigure 4: Crude survival curves for time to inflammatory bowel disease, stratified by exposure group
Estimated with Kaplan-Meier with pointwise confidence intervals from bootstrapping.
IBD=Inflammatory Bowel Disease

© 2026 Kallin S et al. *JAMA Neurology*.

Sensitivity Analysis – Ever-treated approach to follow-up

To address the differences in time on treatment we used an ever-treated approach to follow-up, meaning that only the first occurrence of each exposure per person was included and follow-up did not end at therapy discontinuation.

eTable 6: Observed time, events, and incidence rates, stratified by exposure group

Group	N	Person-years (median)	Events	CD/UC/Unclassified (%)	IR (Crude)
Rituximab	6,331	42,267 (6.8)	45	28/7/10 (62/16/22)	106.5 (77.9–140.5)
Comparator DMTs	6,368	56,248 (9.8)	30	14/7/9 (47/23/30)	53.3 (33.7–74.5)
General Population	56,271	440,925 (8.3)	193	56/113/24 (29/59/12)	43.8 (36.2–51.8)
Natalizumab	2,459	19,780 (8.6)	12	5/3/4 (42/25/33)	60.7 (29.9–97.7)
Dimethyl Fumarate	2,476	20,768 (9.3)	12	5/2/5 (42/17/42)	57.8 (28.3–94.1)
Fingolimod	1,433	15,699 (11.5)	6	4/2/0 (67/33/0)	38.2 (12.4–71.2)

Incidence rates (IR) per 100,000 person-years. Comparator DMTs grouped natalizumab, dimethyl fumarate, and fingolimod. CD=Crohn's Disease; UC=Ulcerative Colitis

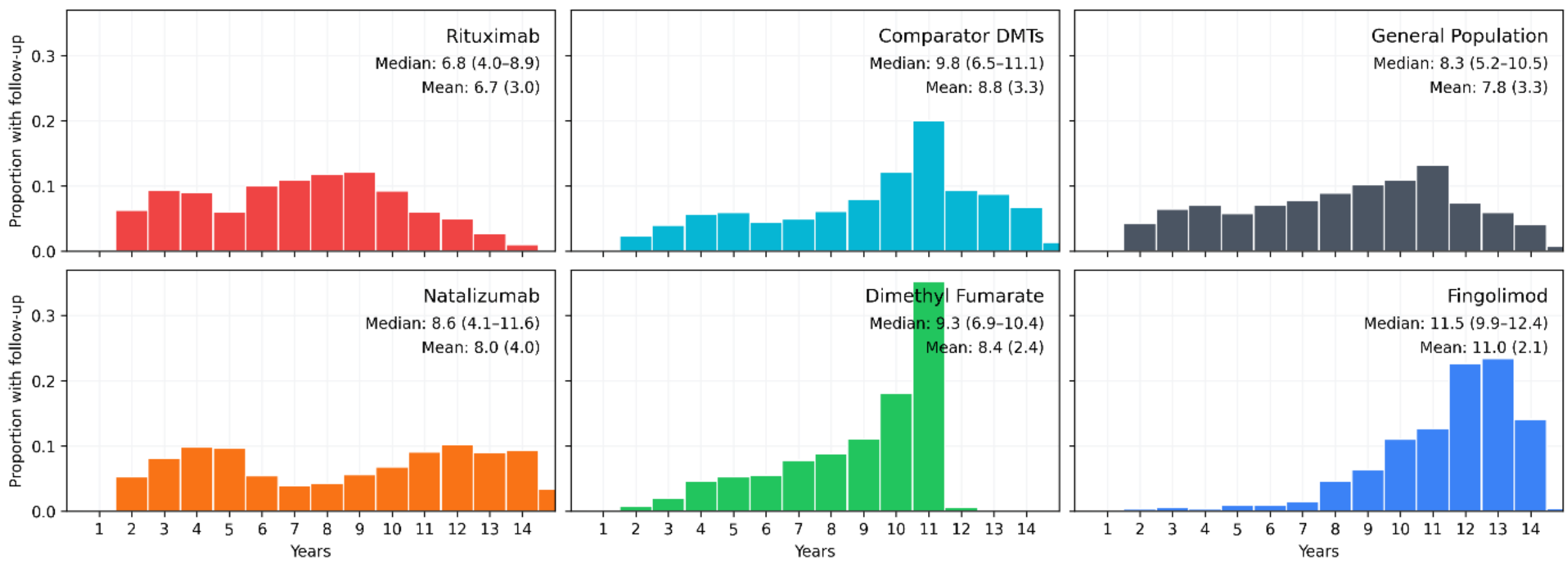

eFigure 5: Distribution of follow-up time stratified by exposure
Comparator disease-modifying therapies (DMTs) grouped natalizumab, dimethyl fumarate, and fingolimod.

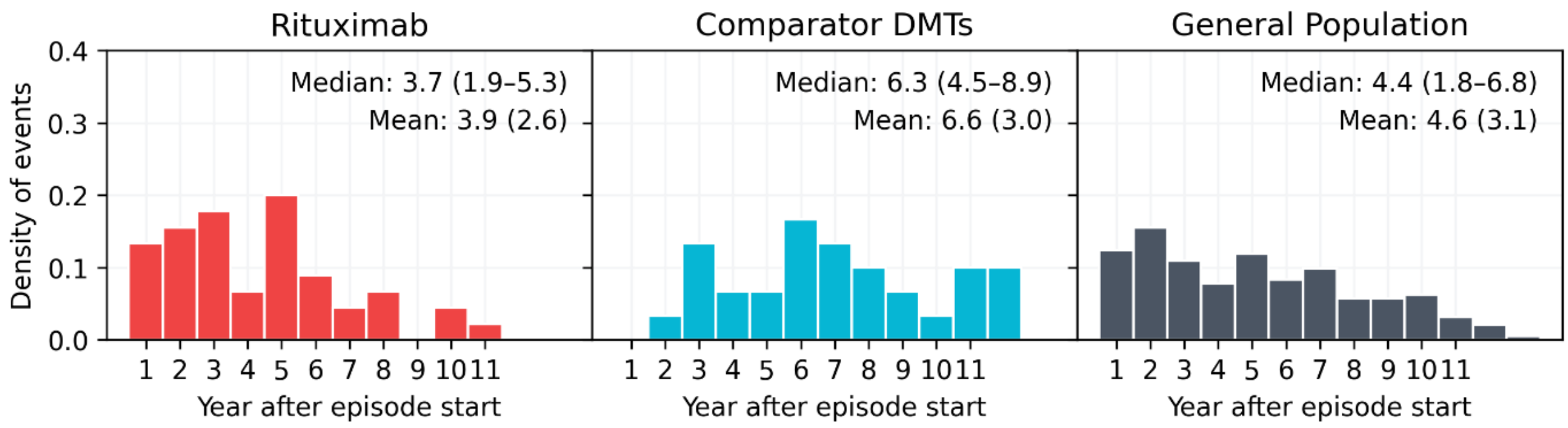

eFigure 6: Distribution of time to outcome, stratified by exposure
Comparator disease-modifying therapies (DMTs) grouped natalizumab, dimethyl fumarate, and fingolimod.

© 2026 Kallin S et al. *JAMA Neurology*.

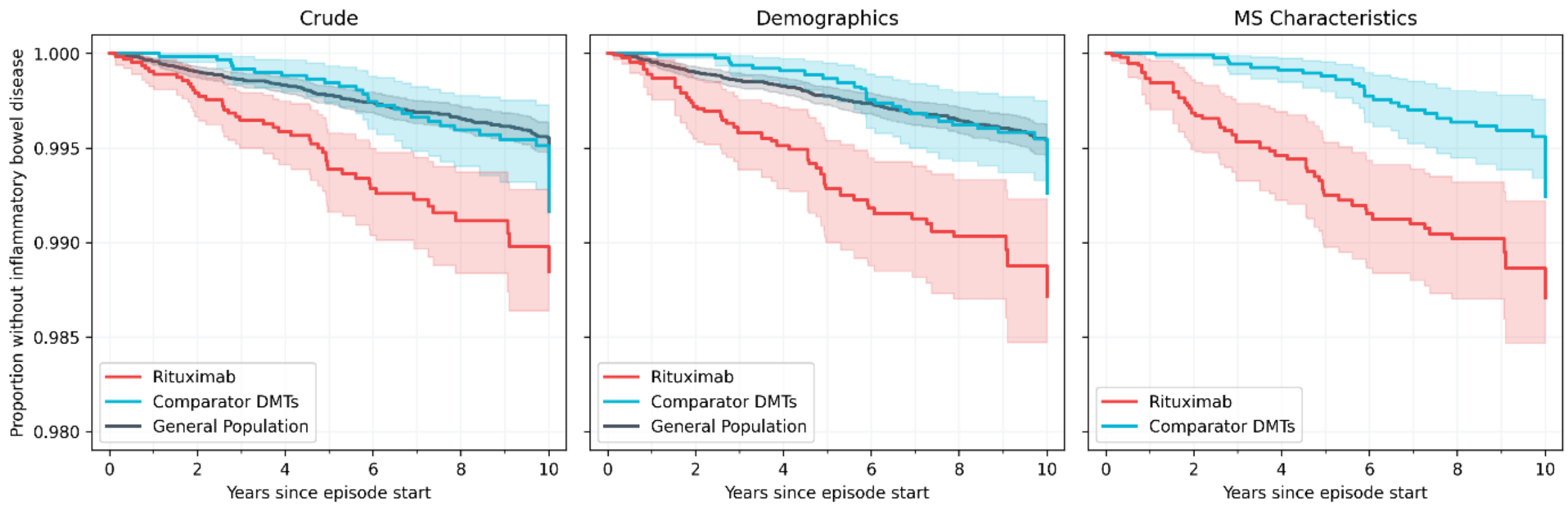

Years since episode start	0	1	2	3	4	5	6	7	8	9	10
Rituximab	6,331 (100)	6,319 (100)	5,921 (94)	5,327 (84)	4,761 (75)	4,377 (69)	3,744 (59)	3,052 (48)	2,305 (36)	1,534 (24)	948 (15)
Comparator DMTs	6,368 (100)	6,363 (100)	6,208 (97)	5,951 (93)	5,593 (88)	5,210 (82)	4,926 (77)	4,614 (72)	4,224 (66)	3,719 (58)	2,947 (46)
General Population	56,271 (100)	56,191 (100)	53,795 (96)	50,153 (89)	46,204 (82)	42,942 (76)	38,951 (69)	34,588 (61)	29,566 (53)	23,856 (42)	17,688 (31)

eFigure 7: Crude and adjusted survival curves for time to inflammatory bowel disease, stratified by exposure group

Estimated with Kaplan-Meier with pointwise confidence intervals from bootstrapping. Numbers at risk stratified by group and year are shown below the plots. Comparator DMTs grouped natalizumab, dimethyl fumarate, and fingolimod. Demographics: adjusted for sex, age, country of birth (Sweden or other), civil status (married/partner), annual income, education, and area of residence in Sweden. MS Characteristics: adjusted for MS duration, year of therapy start, number of previous DMTs, number of previous relapses, and baseline Expanded Disability Status Scale (EDSS), Symbol Digit Modalities Test (SDMT), and Multiple Sclerosis Impact Scale (MSIS-29).

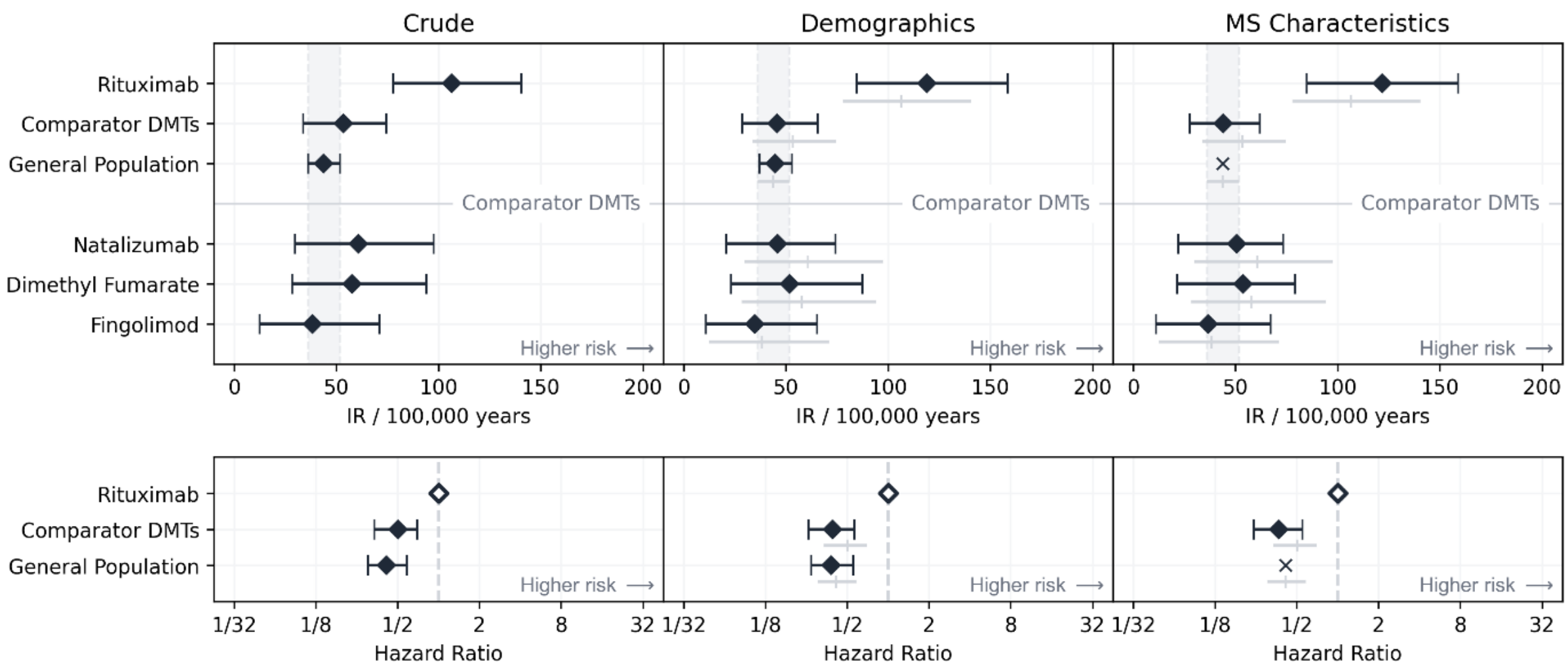

eFigure 8: Forest plots of crude and adjusted incidence rates and hazard ratios of inflammatory bowel disease, stratified by exposure group

Penalized hazard ratios with time since episode start as the time scale and rituximab as the reference. Comparator DMTs grouped natalizumab, dimethyl fumarate, and fingolimod. For the adjusted incidence rates (IRs), separate models were fitted with the comparator DMTs grouped (upper rows) and ungrouped (lower rows). Hazard ratios (HRs) were not estimated for the ungrouped therapies due to sparse data. The crude IR span for the general population is indicated in all IR plots and the crude estimates are indicated in all adjusted plots. Demographics: adjusted for sex, age, country of birth (Sweden or other), civil status (married/partner), annual income, education, and area of residence in Sweden. MS Characteristics: adjusted for MS duration, year of therapy start, number of previous DMTs, number of previous relapses, and baseline Expanded Disability Status Scale (EDSS), Symbol Digit Modalities Test (SDMT), and Multiple Sclerosis Impact Scale (MSIS-29).

© 2026 Kallin S et al. *JAMA Neurology*.

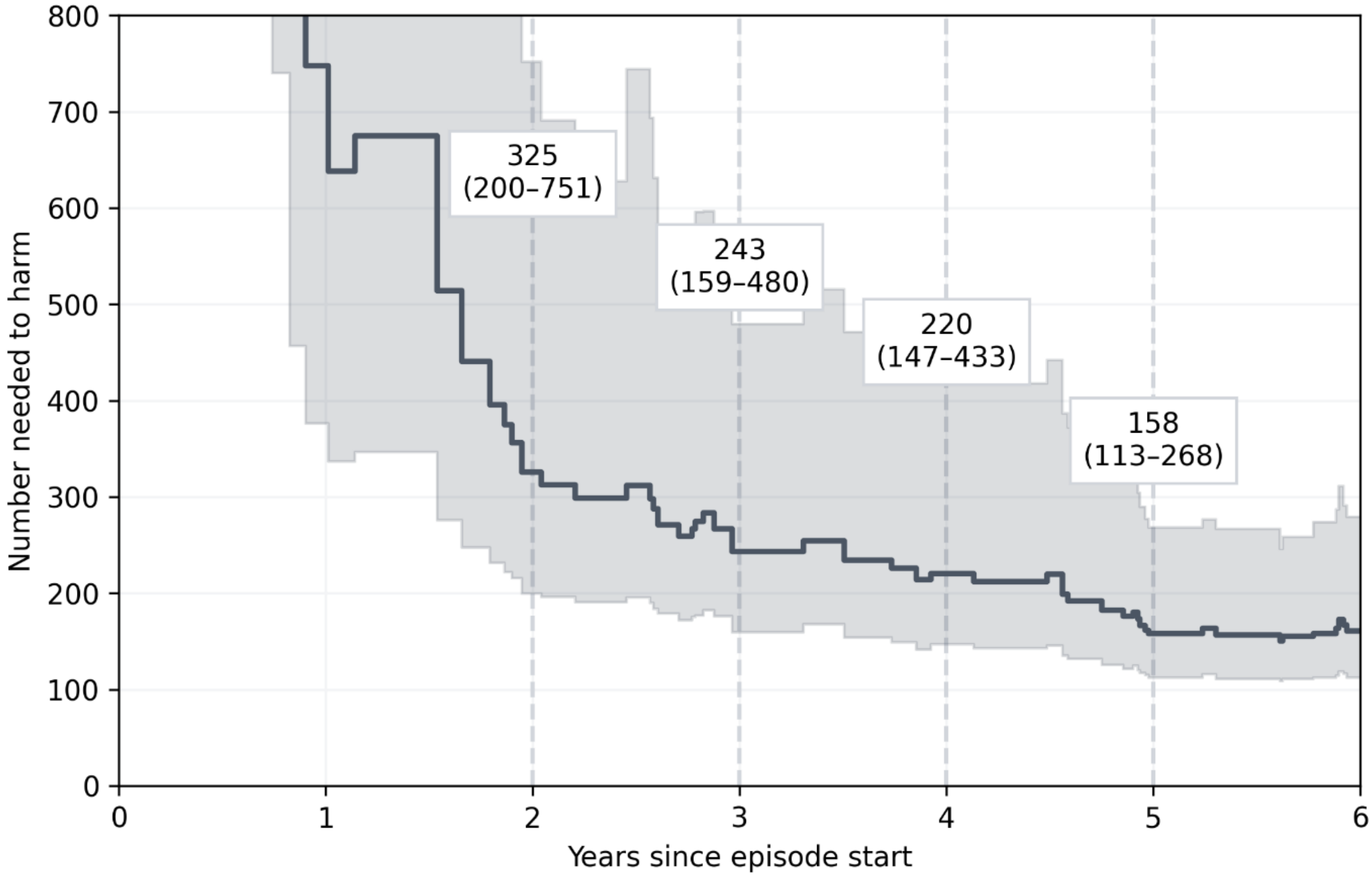

eFigure 9: Time-specific numbers needed to harm with rituximab vs. the comparator DMTs in the fully adjusted model

Comparator DMTs grouped natalizumab, dimethyl fumarate, and fingolimod. Adjusted for: sex, age, country of birth (Sweden or other), civil status (married/partner), annual income, education, area of residence in Sweden, MS duration, year of therapy start, number of previous DMTs, number of previous relapses, and baseline Expanded Disability Status Scale (EDSS), Symbol Digit Modalities Test (SDMT), and Multiple Sclerosis Impact Scale (MSIS-29).

© 2026 Kallin S et al. *JAMA Neurology*.

Sensitivity Analysis – Stricter IBD definition

To address potential misclassification of the outcome, we used a stricter definition of IBD, requiring at least three observations on separate dates in inpatient or secondary outpatient care, with the code K50 (Crohn's disease), K51 (ulcerative colitis), and/or K52.3 (indeterminate colitis) and at least one year between the first and the last observation. To allow for enough follow-up to support this definition, we restricted the end of the inclusion period to 2020-12-31, allowing for at least five years of follow-up. This stricter outcome definition aimed to test the sensitivity of the validated main definition to misclassification of non-IBD colitis or temporary colitis adverse events.

eTable 7: Observed time, events, and incidence rates, stratified by exposure group

Group	N	Person-years (median)	Events	CD/UC/Unclassified (%)	IR (Crude)
Rituximab	4,775	31,728 (6.7)	30	19/4/7 (63/13/23)	94.6 (65.1–128.2)
Comparator DMTs	6,263	28,022 (3.1)	4	1/3/0 (25/75/0)	14.3 (3.5–28.9)
General Population	49,402	268,373 (5.4)	78	25/43/10 (32/55/13)	29.1 (22.8–36.2)
Natalizumab	2,241	9,896 (2.9)	2	1/1/0 (50/50/0)	20.2 (0.0–50.4)
Dimethyl Fumarate	2,518	9,951 (2.8)	1	0/1/0 (0/100/0)	10.0 (0.0–31.8)
Fingolimod	1,504	8,175 (4.3)	1	0/1/0 (0/100/0)	12.2 (0.0–38.4)

Incidence rates (IR) per 100,000 person-years. Comparator DMTs grouped natalizumab, dimethyl fumarate, and fingolimod. CD=Crohn's Disease; UC=Ulcerative Colitis

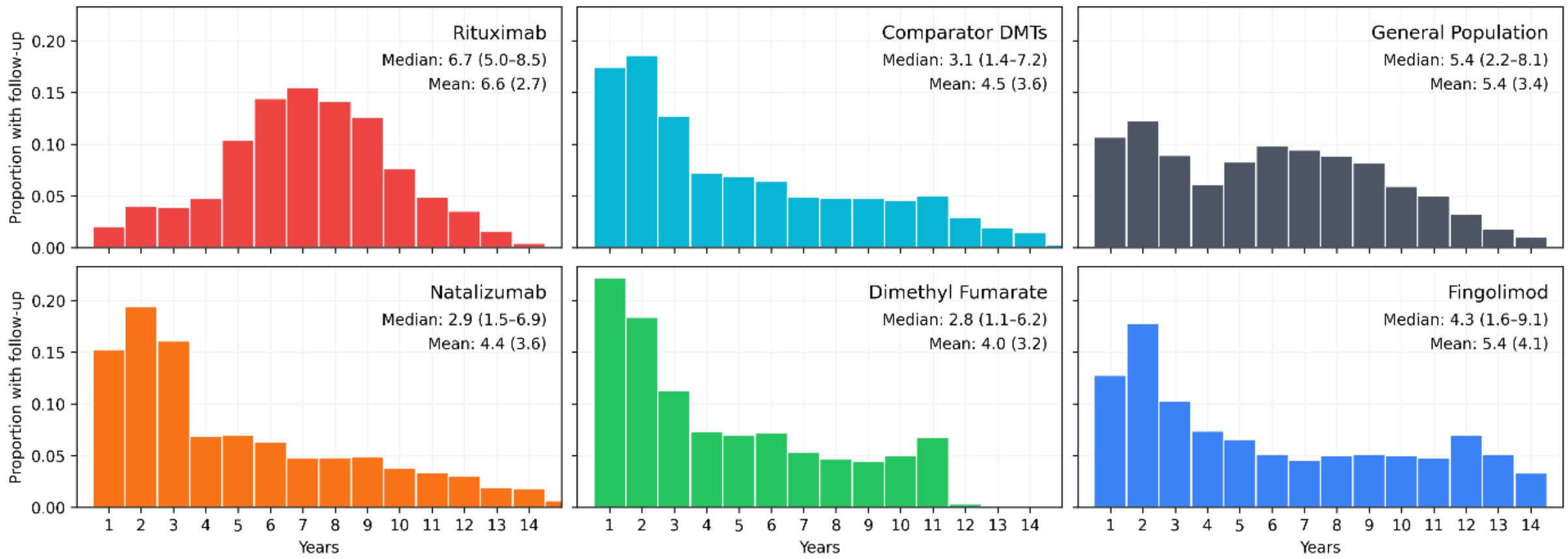

eFigure 10: Distribution of follow-up time stratified by exposure
Comparator disease-modifying therapies (DMTs) grouped natalizumab, dimethyl fumarate, and fingolimod.

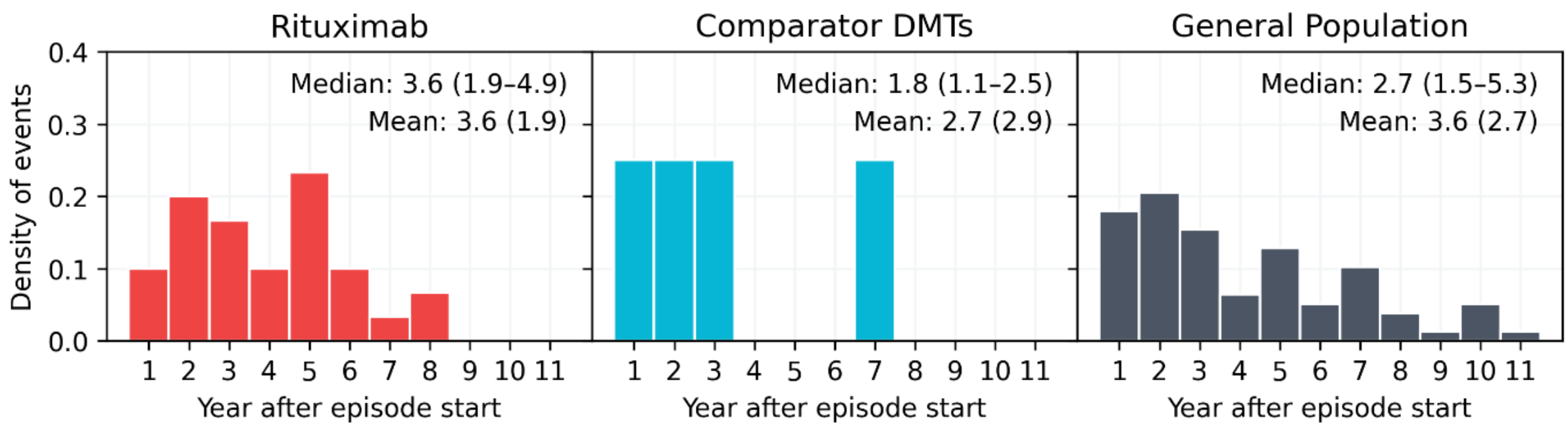

eFigure 11: Distribution of time to outcome, stratified by exposure
Comparator disease-modifying therapies (DMTs) grouped natalizumab, dimethyl fumarate, and fingolimod.

© 2026 Kallin S et al. *JAMA Neurology*.

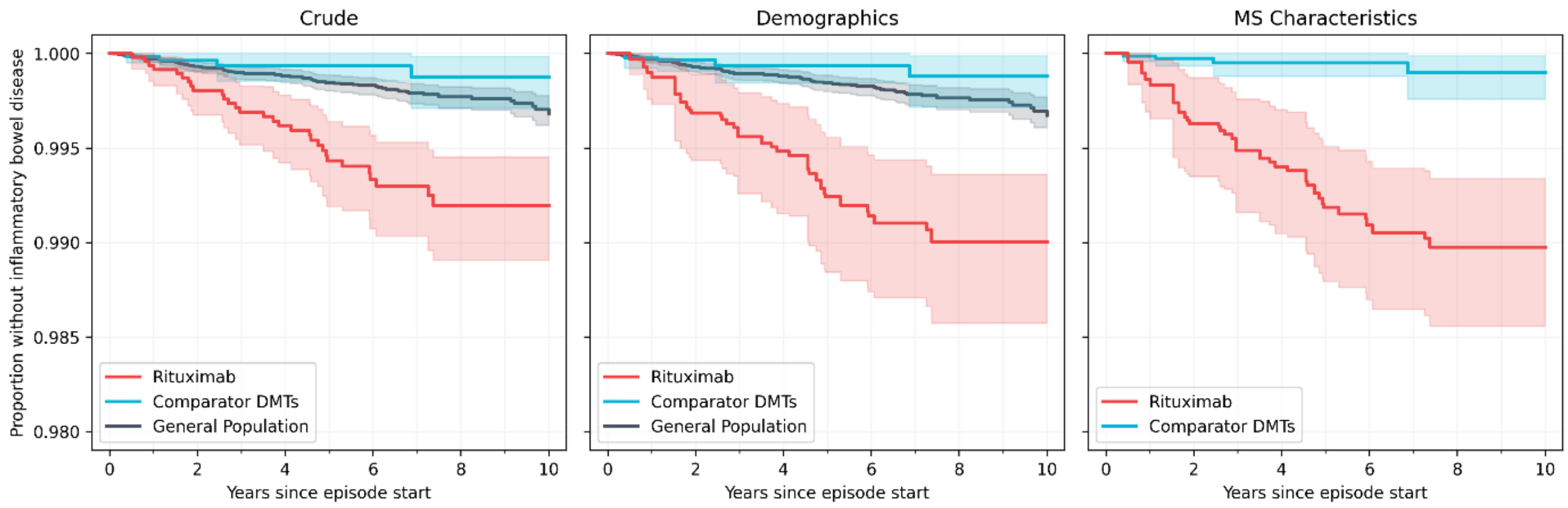

Years since episode start	0	1	2	3	4	5	6	7	8	9	10
Rituximab	4,775 (100)	4,679 (98)	4,488 (94)	4,300 (90)	4,074 (85)	3,574 (75)	2,886 (60)	2,148 (45)	1,473 (31)	870 (18)	506 (11)
Comparator DMTs	6,263 (100)	5,172 (83)	4,008 (64)	3,210 (51)	2,760 (44)	2,327 (37)	1,926 (31)	1,616 (26)	1,314 (21)	1,014 (16)	728 (12)
General Population	49,402 (100)	44,127 (89)	38,041 (77)	33,631 (68)	30,625 (62)	26,524 (54)	21,679 (44)	17,003 (34)	12,629 (26)	8,558 (17)	5,626 (11)

eFigure 12: Crude and adjusted survival curves for time to inflammatory bowel disease, stratified by exposure group
Estimated with Kaplan-Meier with pointwise confidence intervals from bootstrapping. Numbers at risk stratified by group and year are shown below the plots. Comparator DMTs grouped natalizumab, dimethyl fumarate, and fingolimod. Demographics: adjusted for sex, age, country of birth (Sweden or other), civil status (married/partner), annual income, education, and area of residence in Sweden. MS Characteristics: adjusted for MS duration, year of therapy start, number of previous DMTs, number of previous relapses, and baseline Expanded Disability Status Scale (EDSS), Symbol Digit Modalities Test (SDMT), and Multiple Sclerosis Impact Scale (MSIS-29).

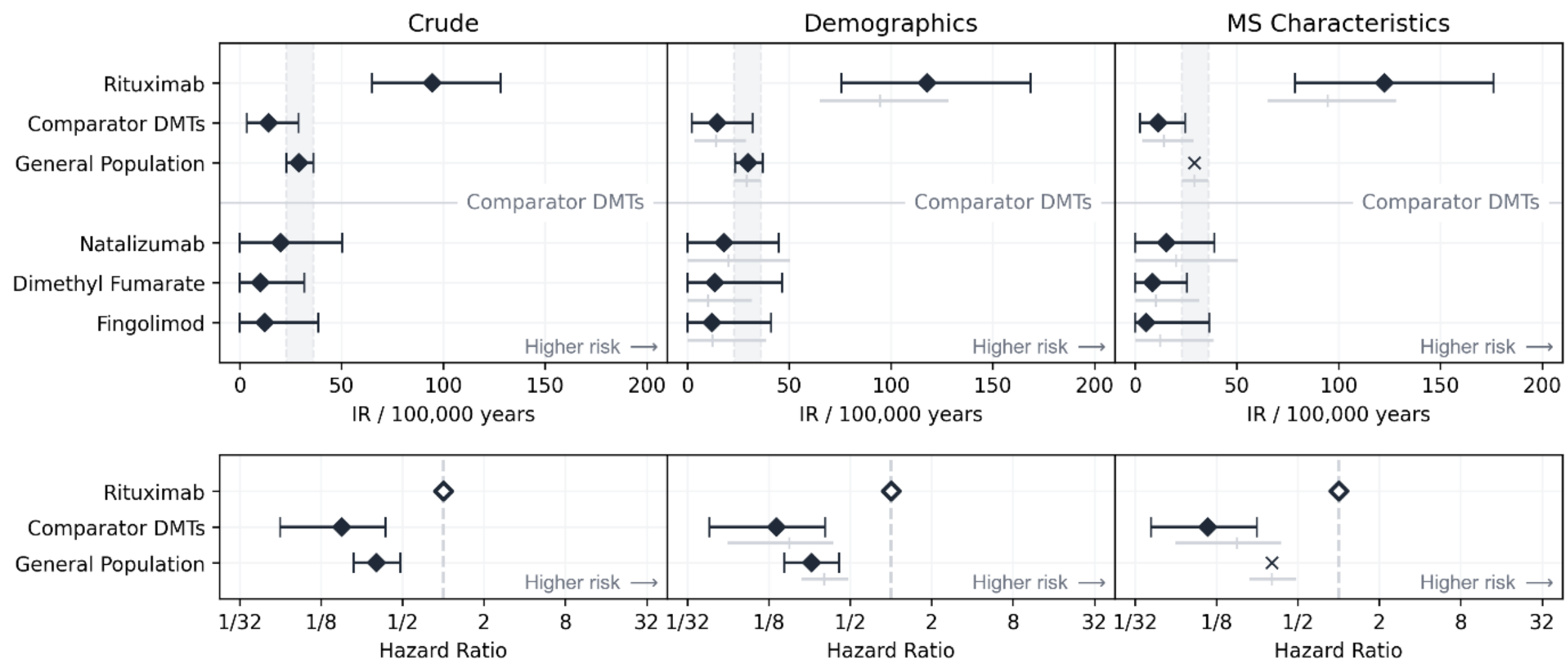

eFigure 13: Forest plots of crude and adjusted incidence rates and hazard ratios of inflammatory bowel disease, stratified by exposure group
Penalized hazard ratios with time since episode start as the time scale and rituximab as the reference. Comparator DMTs grouped natalizumab, dimethyl fumarate, and fingolimod. For the adjusted incidence rates (IRs), separate models were fitted with the comparator DMTs grouped (upper rows) and ungrouped (lower rows). Hazard ratios (HRs) were not estimated for the ungrouped therapies due to sparse data. The crude IR span for the general population is indicated in all IR plots and the crude estimates are indicated in all adjusted plots. Demographics: adjusted for sex, age, country of birth (Sweden or other), civil status (married/partner), annual income, education, and area of residence in Sweden. MS Characteristics: adjusted for MS duration, year of therapy start, number of previous DMTs, number of previous relapses, and baseline Expanded Disability Status Scale (EDSS), Symbol Digit Modalities Test (SDMT), and Multiple Sclerosis Impact Scale (MSIS-29).

© 2026 Kallin S et al. *JAMA Neurology*.

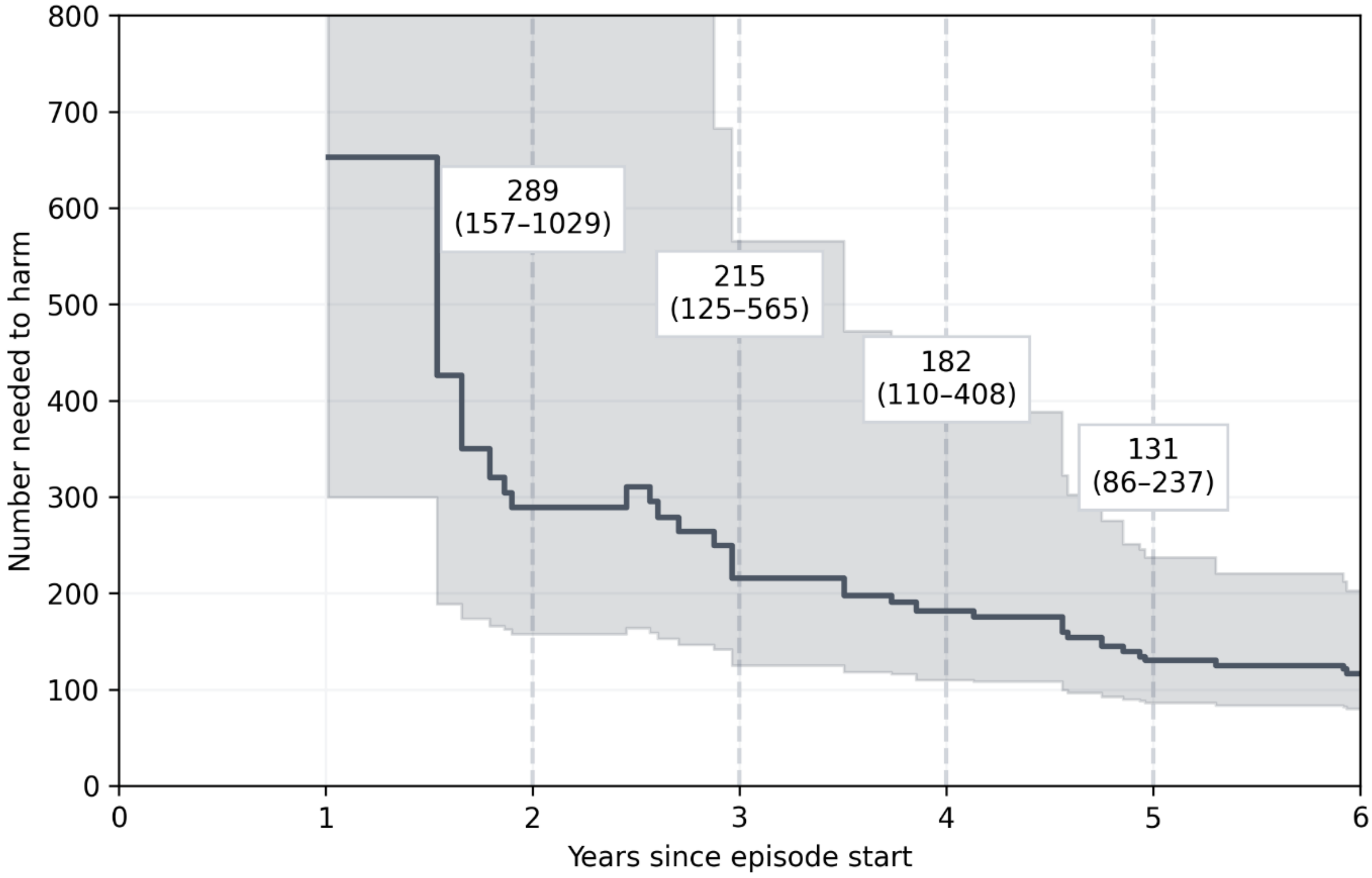

eFigure 14: Time-specific numbers needed to harm with rituximab vs. the comparator DMTs in the fully adjusted model

Comparator DMTs grouped natalizumab, dimethyl fumarate, and fingolimod. Adjusted for: sex, age, country of birth (Sweden or other), civil status (married/partner), annual income, education, area of residence in Sweden, MS duration, year of therapy start, number of previous DMTs, number of previous relapses, and baseline Expanded Disability Status Scale (EDSS), Symbol Digit Modalities Test (SDMT), and Multiple Sclerosis Impact Scale (MSIS-29).

© 2026 Kallin S et al. *JAMA Neurology*.

Sensitivity Analysis – Restricted inclusion period

To address that treatment patterns have changed over time, possibly coinciding with changes in intensity of or guidelines for IBD diagnosis, we restricted the inclusion to a six-year period (2013–2018) with higher overlap between the therapy groups.

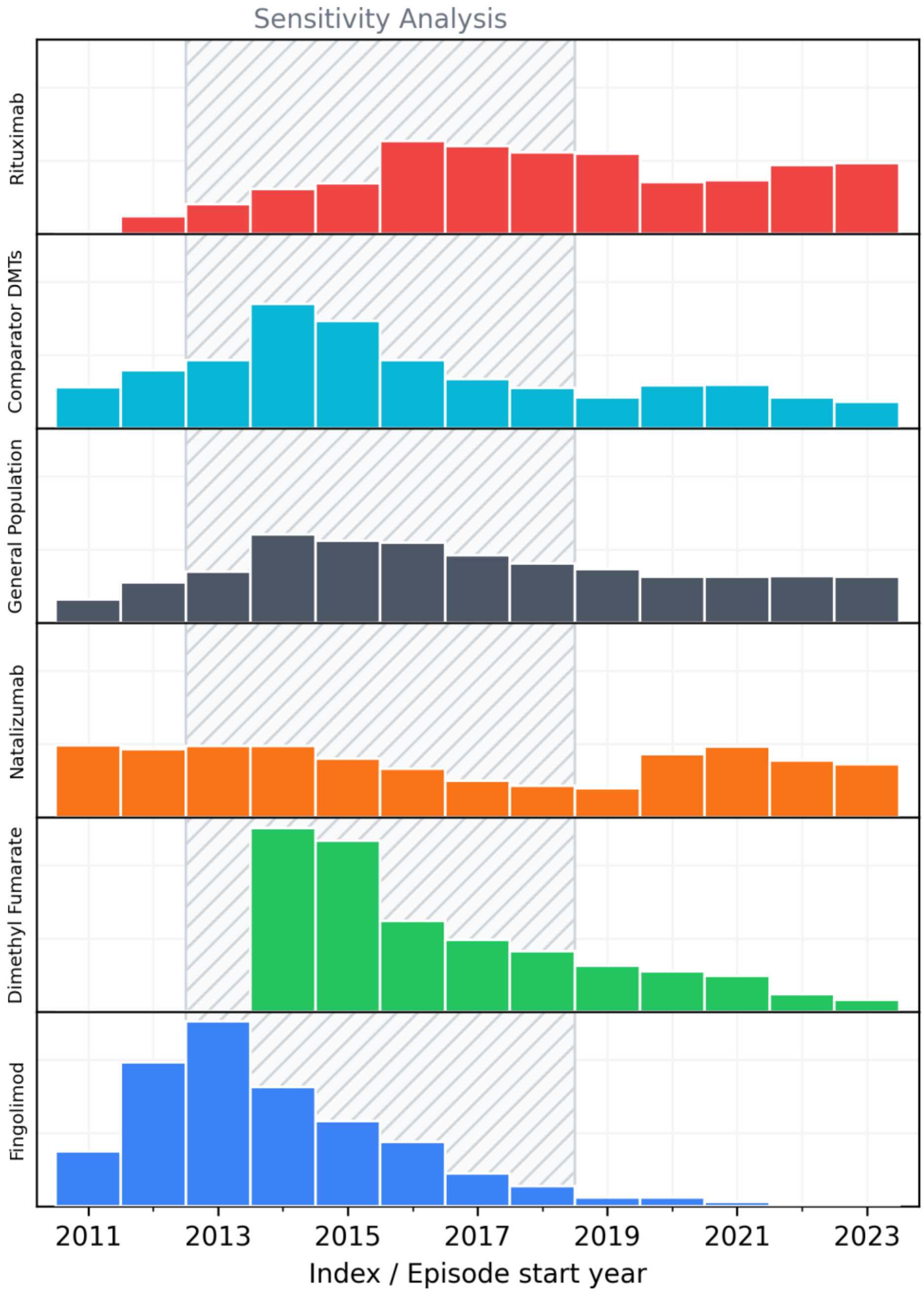

eFigure 15: Distributions of index (episode start) year in the main analysis, stratified by exposure group, with the six-year period selected for sensitivity analysis indicated
Comparator disease-modifying therapies (DMTs) grouped natalizumab, dimethyl fumarate, and fingolimod.

© 2026 Kallin S et al. *JAMA Neurology*.

eTable 8: Observed time, events, and incidence rates, stratified by exposure group

Group	N	Person-years (median)	Events	CD/UC/Unclassified (%)	IR (Crude)
Rituximab	3,431	24,352 (7.4)	30	18/4/8 (60/13/27)	123.2 (82.3–168.9)
Comparator DMTs	4,541	20,413 (3.1)	4	1/3/0 (25/75/0)	19.6 (4.8–40.1)
General Population	35,760	201,496 (6.3)	80	23/42/15 (29/52/19)	39.7 (31.1–48.7)
Natalizumab	1,296	6,014 (3.3)	2	1/1/0 (50/50/0)	33.3 (0.0–85.0)
Dimethyl Fumarate	2,190	8,856 (2.7)	1	0/1/0 (0/100/0)	11.3 (0.0–34.7)
Fingolimod	1,055	5,543 (4.1)	1	0/1/0 (0/100/0)	18.0 (0.0–56.2)

Incidence rates (IR) per 100,000 person-years. Comparator DMTs grouped natalizumab, dimethyl fumarate, and fingolimod. CD=Crohn's Disease; UC=Ulcerative Colitis

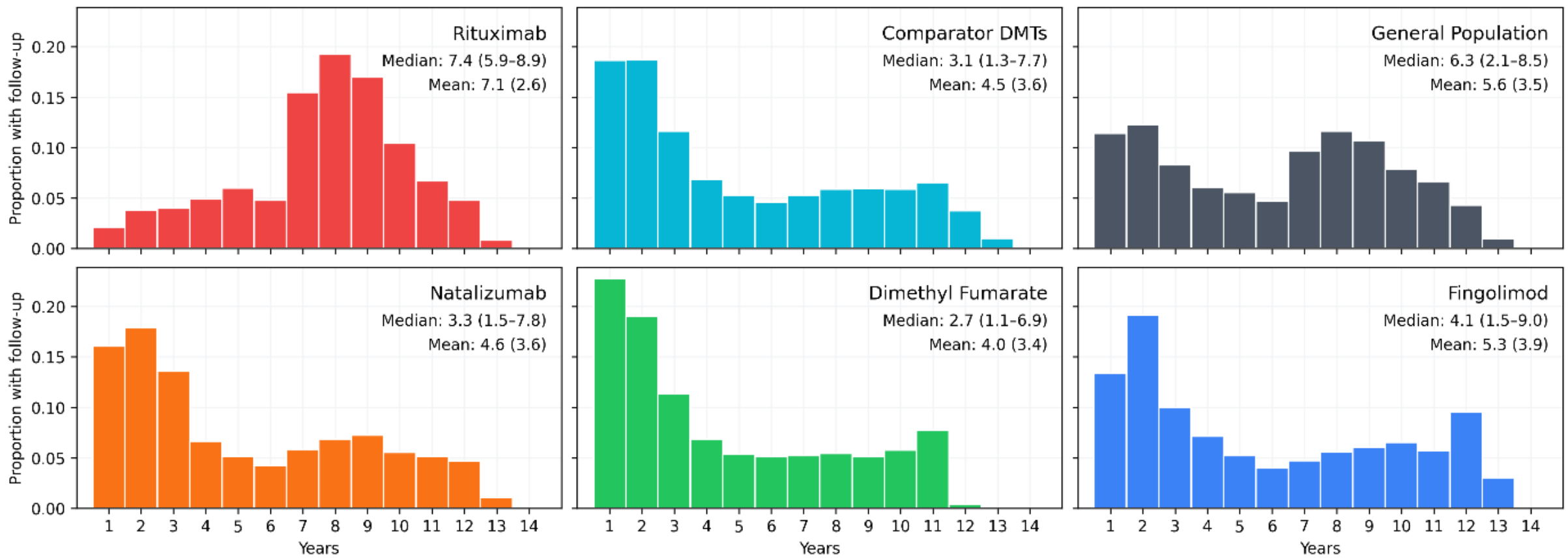

eFigure 16: Distribution of follow-up time stratified by exposure
Comparator disease-modifying therapies (DMTs) grouped natalizumab, dimethyl fumarate, and fingolimod.

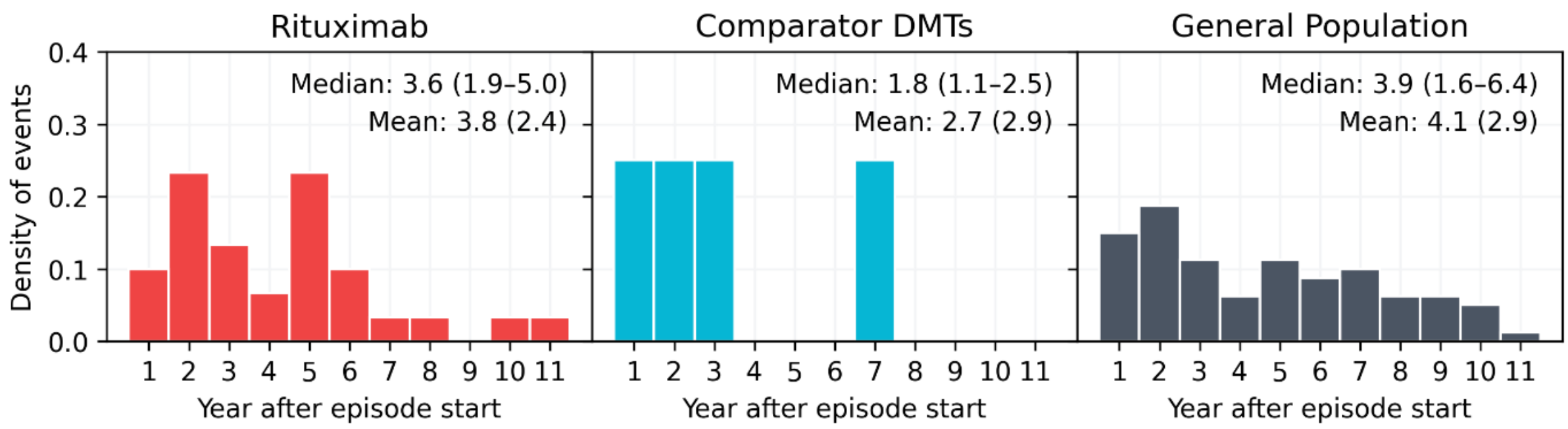

eFigure 17: Distribution of time to outcome, stratified by exposure
Comparator disease-modifying therapies (DMTs) grouped natalizumab, dimethyl fumarate, and fingolimod.

© 2026 Kallin S et al. *JAMA Neurology*.

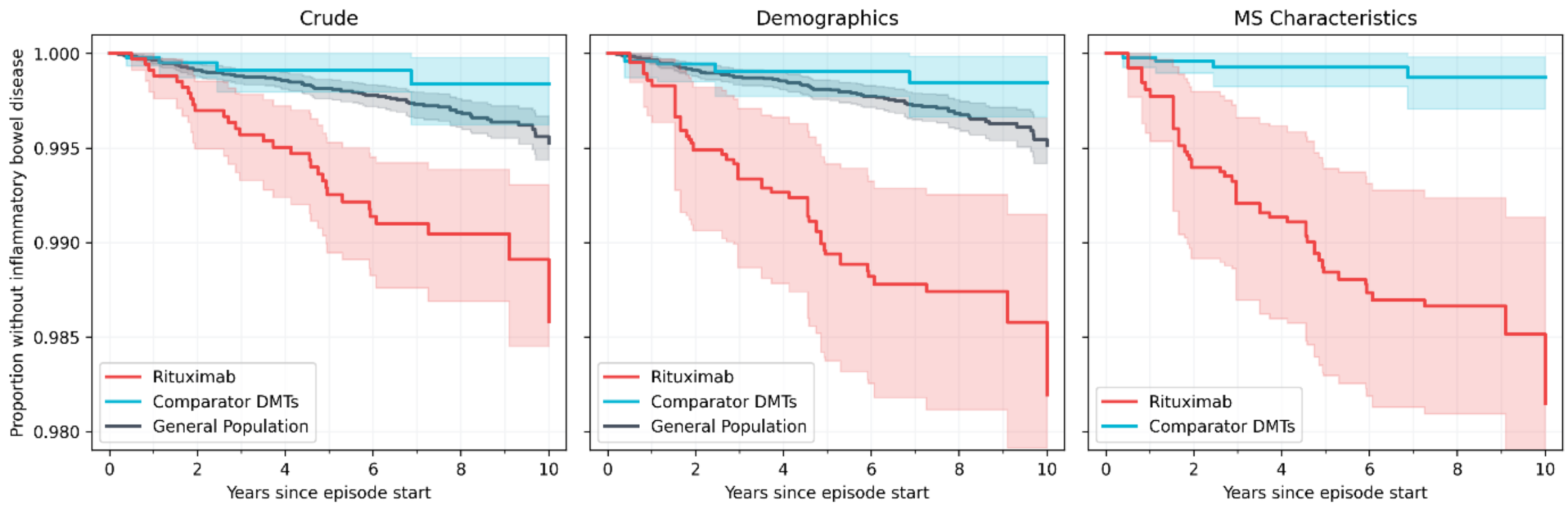

Years since episode start	0	1	2	3	4	5	6	7	8	9	10
Rituximab	3,431 (100)	3,360 (98)	3,229 (94)	3,092 (90)	2,923 (85)	2,717 (79)	2,553 (74)	2,022 (59)	1,361 (40)	779 (23)	422 (12)
Comparator DMTs	4,541 (100)	3,694 (81)	2,844 (63)	2,315 (51)	2,004 (44)	1,765 (39)	1,556 (34)	1,317 (29)	1,051 (23)	780 (17)	513 (11)
General Population	35,760 (100)	31,686 (89)	27,287 (76)	24,323 (68)	22,172 (62)	20,186 (56)	18,505 (52)	15,053 (42)	10,898 (30)	7,070 (20)	4,261 (12)

eFigure 18: Crude and adjusted survival curves for time to inflammatory bowel disease, stratified by exposure group
Estimated with Kaplan-Meier with pointwise confidence intervals from bootstrapping. Numbers at risk stratified by group and year are shown below the plots. Comparator DMTs grouped natalizumab, dimethyl fumarate, and fingolimod. Demographics: adjusted for sex, age, country of birth (Sweden or other), civil status (married/partner), annual income, education, and area of residence in Sweden. MS Characteristics: adjusted for MS duration, year of therapy start, number of previous DMTs, number of previous relapses, and baseline Expanded Disability Status Scale (EDSS), Symbol Digit Modalities Test (SDMT), and Multiple Sclerosis Impact Scale (MSIS-29).

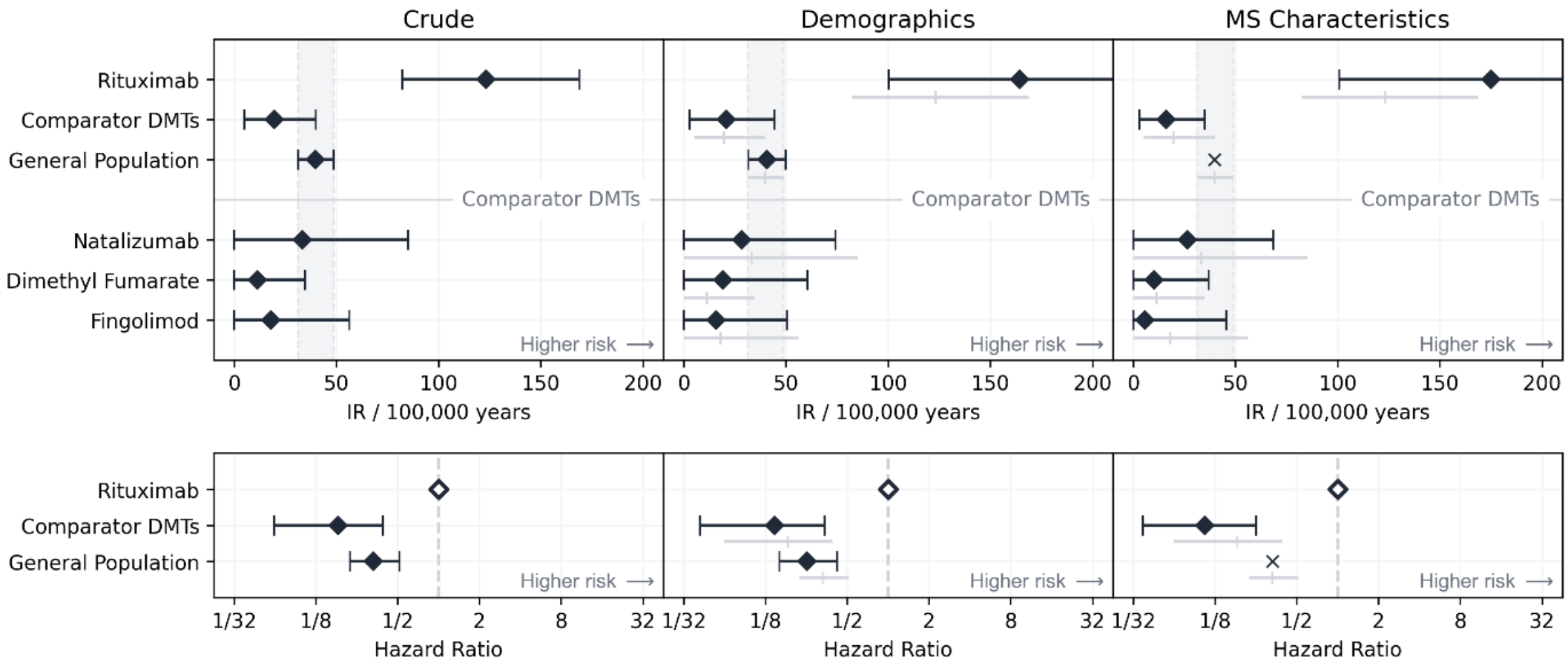

eFigure 19: Forest plots of crude and adjusted incidence rates and hazard ratios of inflammatory bowel disease, stratified by exposure group
Penalized hazard ratios with time since episode start as the time scale and rituximab as the reference. Comparator DMTs grouped natalizumab, dimethyl fumarate, and fingolimod. For the adjusted incidence rates (IRs), separate models were fitted with the comparator DMTs grouped (upper rows) and ungrouped (lower rows). Hazard ratios (HRs) were not estimated for the ungrouped therapies due to sparse data. The crude IR span for the general population is indicated in all IR plots and the crude estimates are indicated in all adjusted plots. Demographics: adjusted for sex, age, country of birth (Sweden or other), civil status (married/partner), annual income, education, and area of residence in Sweden. MS Characteristics: adjusted for MS duration, year of therapy start, number of previous DMTs, number of previous relapses, and baseline Expanded Disability Status Scale (EDSS), Symbol Digit Modalities Test (SDMT), and Multiple Sclerosis Impact Scale (MSIS-29).

© 2026 Kallin S et al. *JAMA Neurology*.

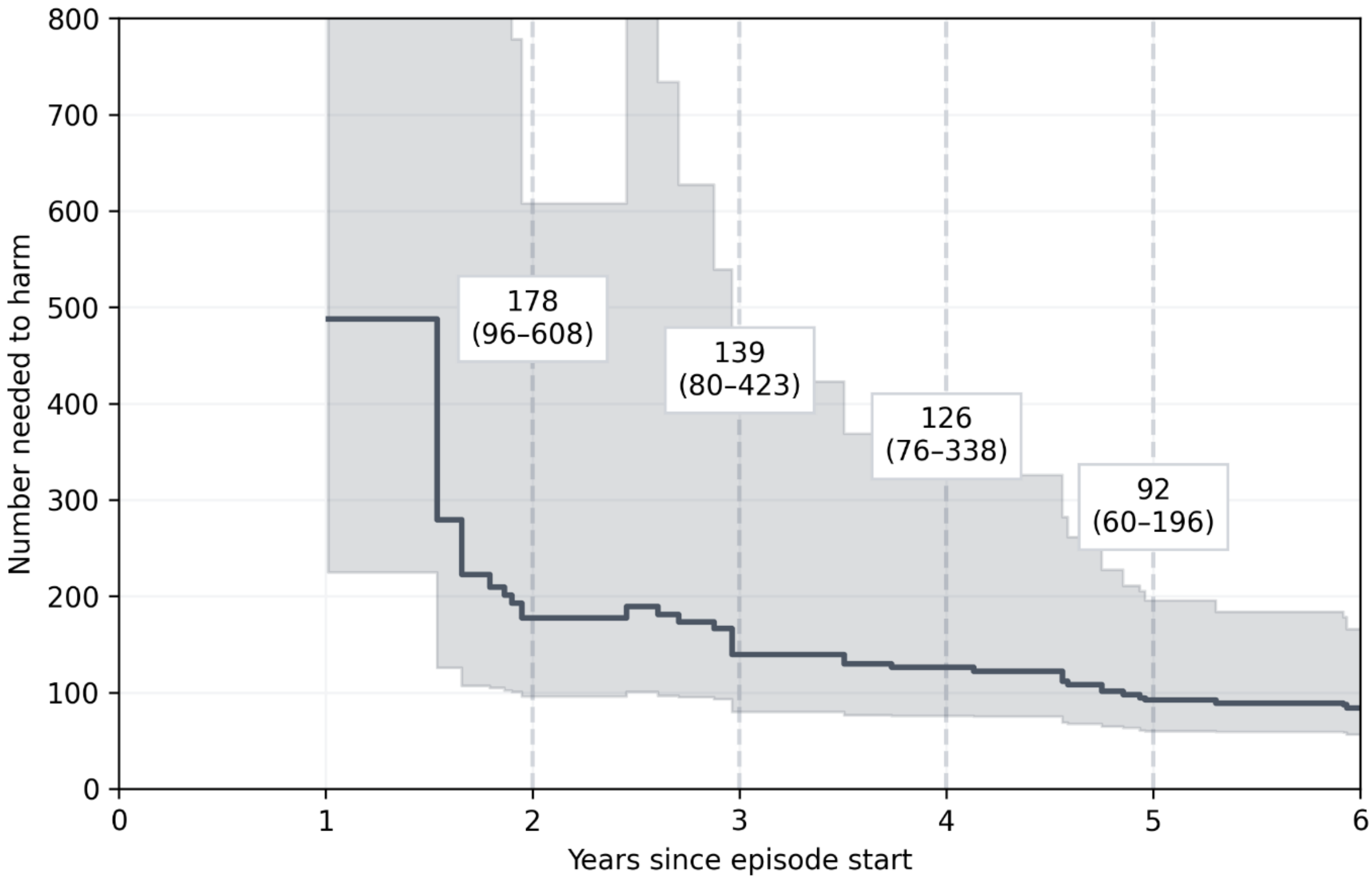

eFigure 20: Time-specific numbers needed to harm with rituximab vs. the comparator DMTs in the fully adjusted model

Comparator DMTs grouped natalizumab, dimethyl fumarate, and fingolimod. Adjusted for: sex, age, country of birth (Sweden or other), civil status (married/partner), annual income, education, area of residence in Sweden, MS duration, year of therapy start, number of previous DMTs, number of previous relapses, and baseline Expanded Disability Status Scale (EDSS), Symbol Digit Modalities Test (SDMT), and Multiple Sclerosis Impact Scale (MSIS-29).

© 2026 Kallin S et al. *JAMA Neurology*.
