# Supplementary material for: Risk of Inflammatory Bowel Disease After Anti-CD20 Treatment in Multiple Sclerosis
Source: JAMA Neurol. 2026 Oct 22:e263439. Online ahead of print. doi: 10.1001/jamaneurol.2026.3439 (PMC13691918; doi:10.1001/jamaneurol.2026.3439)
Supplement: Supplementary file 2 [file jamaneurol-e263439-s002.pdf]

Data Sharing Statement

Kallin. Risk of Inflammatory Bowel Disease After Anti-CD20 Treatment in Multiple Sclerosis. *JAMA Neurol*. Published October 22, 2026. doi:10.1001/jamaneurol.2026.3439

Data
Data available: Yes
Data types: Other (please specify)
Additional Information: The data used in this study constitute sensitive personal information protected by Swedish law and are available from the respective register holders or the corresponding author upon lawful request.
How to access data: Swedish Board of Health and Welfare (Socialstyrelsen), Statistics Sweden (SCB), and/or corresponding author: Peter Alping (peter.alping@ki.se)
When available: With publication

Supporting Documents
Document types: Statistical/analytic code
How to access documents: The analysis code is available from the corresponding author upon request.
When available: With publication

Additional Information
Who can access the data: Data will be provided upon request in accordance with Swedish law. Analysis code will be provided to anyone upon reasonable request.
Types of analyses: Data will be made available as permitted by Swedish law regulating the storage and use of sensitive individual-level data.
Mechanisms of data availability: Data will be made available in accordance with Swedish law.
